# Malignant Neoplasms in Mozambique

**DOI:** 10.1038/bjc.1958.21

**Published:** 1958-06

**Authors:** M. D Prates


### MALIGNANT NEOPLASMS IN MOZAMBIQUE

### A Frequency Ratio Survey from 1944–December 31, 1957 and a Comparison with Other Parts of Africa

M. D. PRATES

*From the Hospital Central Miguel Bombarda, Lourenço Marques, Portuguese East Africa*

Received for publication April 2, 1958

During the last 25 years, several reports on the frequency ratio of malignant neoplasms among the African people have appeared from different parts of the African Continent (Pirie, 1921; Smith and Elmes, 1934; Strachan, 1934; Berman, 1935; Vint, 1935; des Ligneris, 1936; Prates, 1938; Elmes and Baldwin, 1947; Gelfand, 1948; Davies, 1948; Findlay, 1949; Higginson, 1951; Thijs, 1957; Wainwright, 1957). Apart from the study on liver cancer and cirrhosis (Prates, 1938), up to the present no information has been forthcoming concerning the cancer frequency ratios in Africans living in the Portuguese province of Mozambique on the East Coast of Africa.

Through the generous assistance of the National Cancer Association of South Africa, a cancer survey unit was established in Lourenço Marques almost two years ago in order to ascertain the risk to cancer for the African population in and around Lourenço Marques. As an essential preliminary to the main survey all the histological material collected in the pathological laboratory during the previous 13 years was analysed. It was hoped that such a preliminary analysis might indicate the kinds of cancers to be expected in this city.

In view of the limited medical services and the paucity of cancer registries in the underdeveloped Continent of Africa, the greatest amount of information about cancer in Africa will continue to be derived, in the immediate future, from post-mortem and hospital statistics. Although these statistics cannot allow any predictions to be made about the risk to cancer for the population as a whole, nevertheless, a comparison of the data accumulated in the hospital at Lourenço Marques over the last 13 years with the preliminary data of the rates survey conducted over a period of 19 months, will afford some indication of the site frequency of certain types of cancers and the potential value which can be attached to the data derived from conventional routine services of pathological laboratories and hospitals in regions where special cancer units and registries are still not available.

Accordingly, it is proposed:

1. To report the kinds of cancers and their frequency ratios in all the material collected in Lourenço Marques during the past 13 years,
2. to compare these date with those accumulated during the 19-month period following the establishment of the Cancer Unit when interest in cancer was greatly stimulated, and
3. to compare the data from Lourenço Marques with those available from other territories in Africa. From this comparison, it will become apparent that although there are some similarities, there are also marked differences in the site frequency of cancer in different parts of Africa suggesting that environmental influences may be operating in predisposing some sites to cancer more than others.

### MATERIAL AND METHODS

The Province of Mozambique is situated between longitude 30° and 42° East and latitude 10° and 27° South and covers 297,731 square miles (771,255 square kilometres). According to the census of 1950, the population comprises 5,647,000 Africans (2,692,863 males and 2,954,137 females); 50,000 Europeans; 26,000 Coloureds; and 15,000 Asiatics. The medical service, including hospitalisation, is provided free for all Africans, for all the other racial groups earning a salary of less than £30 per month and for all civil servants.

TABLE I.—*The Number of In-patients and Out-patients of the Lourenço Marques Hospital for the Year* 1956

| | |
|---|---|
| *In-patients* | |
| Europeans | 3,263 |
| Africans | 13,246 |
| Coloured | 1,258 |
| Total | 17,767 |
| *Out-patients* | |
| Europeans | 26,374 |
| Africans | 47,560 |
| Coloured | 7,629 |
| Total | 81,563 |

The largest hospital in the Province is located in Lourenço Marques and has about 1000 beds for Africans. The in-patients' turnover in 1956 was 13,246 Africans, 3263 Europeans and 1258 Coloureds and Asiatics. Of the female in-patients 5230 were admitted to the maternity wards. If allowance is made for maternity cases, then three-quarters of the in-patients are males, and only one-quarter females.

47,560 Africans, 26,374 Europeans, and 7629 Coloureds and Asiatics sought treatment in the outpatients' department during 1956 (Table I). It should be mentioned that all the pathological material from Mozambique is sent to the hospital " Miguel Bombarda " (Lourenço Morques' hospital) for examination. The greatest number of examinations are conducted for in-patients as well as out-patients in the Lourenço Marques Hospital, and, to a lesser extent, for patients distributed widely throughout the entire province. It follows that the greatest number of cancers are derived from patients treated in and around Lourenço Marques. The material as a whole is more representative of the people inhabiting the South than of those living in the Central and Northern regions.

The rates survey is being conducted on a population of African people numbering approximately 100,000, living in the city and environs of Lourenço Marques.

Prior to the middle of 1956, a total of 1606 malignant neoplasms was diagnosed from autopsy and histological examination. In May 1956, the special survey was initiated. During the past 19 months, a total of 371 malignant tumours was diagnosed by autopsy and histological examination. Tumours diagnosed by any other method, e.g. radiological or clinical, are not considered in the present study. Of the total of 371 tumours, 210 were found in patients not belonging to the population of Lourenço Marques under survey. That is to say, three groups of material were accumulated; the first from 1944 to 1956; the second obtained from all cancer patients seeking treatment at the Lourenço Marques Hospital from May 1956 to December 1957; while the third group included patients with malignant neoplasms belonging strictly to the area under survey from May 1956 to December 1957, inclusive. The total number of tumours recorded in Africans from May 1944 to December 31, 1957 was 1273. In addition, 580 tumours were identified in Europeans and 124 tumours in Coloureds and Asiatics making a total of 1977 malignant neoplasms.

### RESULTS

Table II summarizes the distribution by sex and race of 1977 malignant tumours accumulated over a period of 13 years, and diagnosed at autopsy and by histological examination. Of these tumours 580 were derived from Europeans, 1273 from Africans and 124 from the other races.

Amongst the European males, 303 malignant neoplasms were found. The skin accounted for 30·0 per cent of all tumours and thereafter, in order of frequency, the stomach for 8·9 per cent, the large intestine and rectum together for 8·9 per cent and malignancies of lymphoid tissue (8·9 per cent) and larynx and lung (9·5 per cent) (Table III). Amongst the European females, 23·8 per cent of all carcinomas occurred in the breast, 24·8 per cent in the cervix and the uterus and 15·1 per cent in the skin. That is to say, in females, 63·7 per cent of all cancers were obtained from the breast, uterus (including the cervix) and skin. The ratio of carcinoma of the body of the uterus to carcinoma of the cervix in European women was 1 : 4·6. Three Kaposi tumours were diagnosed in Europeans, two in males and one in a female. One chorionepithelioma, two gliomata of the brain, one glioma of the eye, three sarcomata of the connective tissue and bone and two lymphosarcomata occurred in Europeans under the age of 20 years. Breast carcinoma and carcinoma of the body of the uterus and skin cancer did not appear in European women in Lourenço Marques under the age of 20 years (Table II).

As far as the Africans are concerned, it is evident from Table II that of a total of 842 malignant neoplasms amongst males, 43·1 per cent were primary in the liver. Liver cancer, therefore, was at least three times as common as any other tumour. The skin (13·9 per cent), the lymphatic tumours (8·6 per cent) and the bladder (7·9 per cent) were next in order of frequency. Cancers of these four sites accounted for 73·6 per cent of all cancers in African males as compared with 40·5 per cent for the same sites in European males. Carcinoma of the stomach (1·0 per cent), of the oesophagus (0·4 per cent), colon (0·3 per cent), rectum (0·1 per cent) as well as carcinoma of the larynx (0·2 per cent), lung and bronchus (0·3 per cent) occurred less frequently in the African male than comparable tumours in the European male.

Amongst the African females, carcinoma of the cervix uteri (20·6 per cent), carcinoma of the liver (14·6 per cent), carcinoma of the skin (13·9 per cent) and carcinoma of the bladder (7·2 per cent) comprised 56·3 per cent of all cancers, as compared with 36·6 per cent for tumours in comparable sites in European females.

In the African female, the relative frequency ratio of carcinoma of the breast was 7·4 per cent as compared with 23·8 per cent in the European female. The ratio of carcinoma of the corpus uteri to carcinoma of the cervix uteri was 1 : 18 in African women as compared with 1 : 4·6 in European women. One case of Kaposi's sarcoma was found in an African female as compared with 22 in African males.

TABLE II.—*Distribution of All Malignant Neoplasms by Site, Type and Age in Europeans and Africans from April* 1944–*December* 31, 1957

| No. Int. Nom. | Site | Type | Race | Sex | Age groups 1–10 | 10–20 | 20–30 | 30–40 | 40–50 | 50–60 | Over 60 | ? | Total | Grand Total | Coloured |
|---|---|---|---|---|---|---|---|---|---|---|---|---|---|---|---|
| 140 | Lip | Ca. | Eur. | M. | 0 | 1 | 0 | 0 | 1 | 0 | 5 | 1 | 8 | 18 | |
| | | | | F. | 0 | 0 | 0 | 0 | 0 | 0 | 0 | 0 | 0 | | |
| | | | Afr. | M. | 0 | 1 | 1 | 0 | 1 | 0 | 0 | 0 | 3 | | |
| | | | | F. | 0 | 0 | 3 | 0 | 2 | 0 | 2 | 0 | 7 | | |
| 141 | Tongue | Ca., 10 | Eur. | M. | 0 | 0 | 0 | 0 | 1 | 2 | 2 | 0 | 5 | 9 | + 2 |
| | | | | F. | 0 | 0 | 0 | 0 | 0 | 0 | 0 | 0 | 0 | | |
| | | Sarcoma, 1 | Afr. | M. | 0 | 0 | 1 | 0 | 0 | 3 | 0 | 0 | 4 | | |
| | | | | F. | 0 | 0 | 0 | 0 | 0 | 0 | 0 | 0 | 0 | | |
| 142.1 | Salivary gland Parotid | Ca. | Eur. | M. | 0 | 0 | 1 | 0 | 0 | 0 | 0 | 0 | 1 | 6 | + 1 |
| | | | | F. | 0 | 0 | 0 | 0 | 0 | 0 | 0 | 0 | 0 | | |
| | | | Afr. | M. | 0 | 0 | 0 | 1 | 0 | 0 | 0 | 0 | 1 | | |
| | | | | F. | 0 | 0 | 0 | 1 | 1 | 2 | 1 | 0 | 5 | | |
| 144 | Mouth | Ca. | Eur. | M. | 0 | 0 | 0 | 0 | 0 | 1 | 2 | 1 | 4 | 27 | + 1 |
| | | | | F. | 0 | 0 | 0 | 0 | 0 | 1 | 0 | 0 | 1 | | |
| | | | Afr. | M. | 0 | 0 | 0 | 1 | 5 | 3 | 3 | 1 | 13 | | |
| | | | | F. | 0 | 0 | 0 | 4 | 4 | 0 | 0 | 1 | 9 | | |
| 145A | Tonsil | Ca. | Eur. | M. | 0 | 0 | 0 | 0 | 0 | 1 | 1 | 1 | 3 | 6 | + 2 |
| | | | | F. | 0 | 0 | 0 | 0 | 0 | 0 | 0 | 0 | 0 | | |
| | | | Afr. | M. | 0 | 0 | 0 | 0 | 2 | 1 | 0 | 0 | 3 | | |
| | | | | F. | 0 | 0 | 0 | 0 | 0 | 0 | 0 | 0 | 0 | | |
| 146 | Naso-pharynx | Ca. | Eur. | M. | 0 | 0 | 0 | 0 | 1 | 1 | 0 | 0 | 2 | 3 | |
| | | | | F. | 0 | 0 | 0 | 0 | 0 | 1 | 0 | 0 | 1 | | |
| | | | Afr. | M. | 0 | 0 | 0 | 0 | 0 | 0 | 0 | 0 | 0 | | |
| | | | | F. | 0 | 0 | 0 | 0 | 0 | 0 | 0 | 0 | 0 | | |
| 150 | Oesophagus | Ca. | Eur. | M. | 0 | 0 | 0 | 0 | 0 | 1 | 0 | 0 | 1 | 6 | + 2 |
| | | | | F. | 0 | 0 | 0 | 0 | 1 | 0 | 0 | 0 | 1 | | |
| | | | Afr. | M. | 0 | 1 | 1 | 0 | 1 | 0 | 1 | 0 | 4 | | |
| | | | | F. | 0 | 0 | 0 | 0 | 0 | 0 | 0 | 0 | 0 | | |
| 151 | Stomach | Ca. | Eur. | M. | 0 | 0 | 0 | 2 | 9 | 6 | 10 | 0 | 27 | 49 | + 3 |
| | | | | F. | 0 | 0 | 0 | 1 | 6 | 5 | 1 | 0 | 13 | | |
| | | | Afr. | M. | 0 | 0 | 1 | 0 | 2 | 2 | 4 | 0 | 9 | | |
| | | | | F. | 0 | 0 | 0 | 0 | 0 | 0 | 0 | 0 | 0 | | |
| 152C | Small intestine | Ca. | Eur. | M. | 0 | 0 | 0 | 0 | 0 | 0 | 2 | 0 | 2 | 7 | |
| | | | | F. | 0 | 0 | 0 | 1 | 1 | 0 | 1 | 0 | 3 | | |
| | | | Afr. | M. | 0 | 0 | 0 | 0 | 1 | 0 | 0 | 0 | 1 | | |
| | | | | F. | 0 | 0 | 0 | 0 | 0 | 1 | 0 | 0 | 1 | | |
| 153A | Large intestine | Ca. | Eur. | M. | 0 | 1 | 0 | 1 | 1 | 5 | 9 | 0 | 17 | 26 | + 5 |
| | | | | F. | 0 | 1 | 0 | 1 | 1 | 1 | 2 | 0 | 6 | | |
| | | | Afr. | M. | 0 | 0 | 0 | 1 | 2 | 0 | 0 | 0 | 3 | | |
| | | | | F. | 0 | 0 | 0 | 0 | 0 | 0 | 0 | 0 | 0 | | |

TABLE II—*cont.*

| No. Int. Nom. | Site | Type | Race | Sex | Age groups 1–10 | 10–20 | 20–30 | 30–40 | 40–50 | 50–60 | Over 60 | ? | Total | Grand Total | Coloured |
|---|---|---|---|---|---|---|---|---|---|---|---|---|---|---|---|
| 154 | Rectum | Ca. | Eur. | M. | 0 | 0 | 0 | 0 | 0 | 2 | 7 | 1 | 10 | 16 | + |
| | | | | F. | 0 | 0 | 0 | 0 | 0 | 1 | 1 | 0 | 2 | | |
| | | | Afr. | M. | 0 | 0 | 0 | 0 | 0 | 1 | 0 | 0 | 1 | | |
| | | | | F. | 0 | 0 | 0 | 0 | 0 | 3 | 0 | 0 | 3 | | |
| 155 | Liver | Ca. Primary | Eur. | M. | 0 | 0 | 0 | 0 | 4 | 1 | 3 | 0 | 8 | 437 | + 6 |
| | | | | F. | 0 | 0 | 0 | 0 | 0 | 2 | 1 | 0 | 3 | | |
| | | | Afr. | M. | 2 | 67 | 113 | 83 | 53 | 26 | 15 | 4 | 363 | | |
| | | | | F. | 1 | 7 | 21 | 16 | 7 | 5 | 5 | 1 | 63 | | |
| 155C | Gall bladder | Ca. | Eur. | M. | 0 | 0 | 0 | 0 | 0 | 2 | 2 | 0 | 4 | 12 | + 2 |
| | | | | F. | 0 | 0 | 0 | 1 | 0 | 3 | 2 | 1 | 7 | | |
| | | | Afr. | M. | 0 | 0 | 0 | 1 | 0 | 0 | 0 | 0 | 1 | | |
| | | | | F. | 0 | 0 | 0 | 0 | 0 | 0 | 0 | 0 | 0 | | |
| 157 | Pancreas | Ca. | Eur. | M. | 0 | 0 | 0 | 0 | 1 | 1 | 0 | 1 | 3 | 12 | + 2 |
| | | | | F. | 0 | 0 | 0 | 0 | 0 | 0 | 0 | 0 | 0 | | |
| | | | Afr. | M. | 0 | 0 | 0 | 3 | 3 | 1 | 1 | 0 | 8 | | |
| | | | | F. | 0 | 0 | 0 | 0 | 0 | 0 | 1 | 0 | 1 | | |
| 158 | Abdominal cavity | Ca. | Eur. | M. | 0 | 0 | 0 | 0 | 0 | 2 | 0 | 0 | 2 | 11 | + 1 |
| | | | | F. | 0 | 0 | 0 | 0 | 0 | 1 | 2 | 0 | 3 | | |
| | | | Afr. | M. | 0 | 0 | 1 | 0 | 0 | 1 | 1 | 0 | 3 | | |
| | | | | F. | 0 | 0 | 1 | 1 | 0 | 0 | 1 | 0 | 3 | | |
| 160A | Nose | Ca. | Eur. | M. | 0 | 0 | 0 | 0 | 0 | 2 | 0 | 0 | 2 | 3 | |
| | | | | F. | 0 | 0 | 0 | 0 | 0 | 0 | 0 | 0 | 0 | | |
| | | | Afr. | M. | 0 | 0 | 0 | 1 | 0 | 0 | 0 | 0 | 1 | | |
| | | | | F. | 0 | 0 | 0 | 0 | 0 | 0 | 0 | 0 | 0 | | |
| 161 | Larynx | Ca. | Eur. | M. | 0 | 0 | 0 | 1 | 1 | 5 | 7 | 1 | 15 | 18 | + 2 |
| | | | | F. | 0 | 0 | 0 | 1 | 0 | 0 | 0 | 0 | 1 | | |
| | | | Afr. | M. | 0 | 0 | 0 | 0 | 2 | 0 | 0 | 0 | 2 | | |
| | | | | F. | 0 | 0 | 0 | 0 | 0 | 0 | 0 | 0 | 0 | | |
| 162B 162C | Lung Bronchus | Ca. | Eur. | M. | 0 | 0 | 0 | 0 | 3 | 3 | 7 | 1 | 14 | 18 | + 2 |
| | | | | F. | 0 | 0 | 0 | 0 | 0 | 1 | 0 | 0 | 1 | | |
| | | | Afr. | M. | 0 | 0 | 0 | 0 | 0 | 0 | 3 | 0 | 3 | | |
| | | | | F. | 0 | 0 | 0 | 0 | 0 | 0 | 0 | 0 | 0 | | |
| 170 | Breast | Ca. | Eur. | M. | 0 | 0 | 0 | 0 | 0 | 0 | 0 | 0 | 0 | 99 | + 12 |
| | | | | F. | 0 | 0 | 3 | 12 | 22 | 18 | 8 | 3 | 66 | | |
| | | | Afr. | M. | 0 | 0 | 0 | 0 | 1 | 0 | 0 | 0 | 1 | | |
| | | | | F. | 0 | 0 | 3 | 6 | 9 | 6 | 7 | 1 | 32 | | |
| 171 | Cervix uteri | Ca. | Eur. | M. | 0 | 0 | 0 | 0 | 0 | 0 | 0 | 0 | 0 | 144 | + 21 |
| | | | | F. | 0 | 0 | 0 | 11 | 20 | 14 | 9 | 1 | 55 | | |
| | | | Afr. | M. | 0 | 0 | 0 | 0 | 0 | 0 | 0 | 0 | 0 | | |
| | | | | F. | 0 | 0 | 9 | 18 | 28 | 20 | 10 | 4 | 89 | | |
| 172 | Corpus uteri | Ca., 21 Sarcoma, 2 | Eur. | M. | 0 | 0 | 0 | 0 | 0 | 0 | 0 | 0 | 0 | 18 | + 5 |
| | | | | F. | 0 | 0 | 2 | 1 | 1 | 4 | 5 | 1 | 14 | | |
| | | | Afr. | M. | 0 | 0 | 0 | 0 | 0 | 0 | 0 | 0 | 0 | | |
| | | | | F. | 0 | 0 | 0 | 1 | 1 | 1 | 0 | 1 | 4 | | |
| 173 | Uterus | Chorion-epithelioma | Eur. | M. | 0 | 0 | 0 | 0 | 0 | 0 | 0 | 0 | 0 | 12 | + 4 |
| | | | | F. | 0 | 1 | 0 | 6 | 2 | 0 | 0 | 1 | 10 | | |
| | | | Afr. | M. | 0 | 0 | 0 | 0 | 0 | 0 | 0 | 0 | 0 | | |
| | | | | F. | 0 | 0 | 2 | 0 | 0 | 0 | 0 | 0 | 2 | | |

TABLE II—*cont.*

| No. Int. Nom. | Site | Type | Race | Sex | Age groups 1–10 | 10–20 | 20–30 | 30–40 | 40–50 | 50–60 | Over 60 | ? | Total | Grand Total | Coloured |
|---|---|---|---|---|---|---|---|---|---|---|---|---|---|---|---|
| 175A | Ovary | Ca. | Eur. | M. | 0 | 0 | 0 | 0 | 0 | 0 | 0 | 0 | 0 | 14 | |
| | | | | F. | 0 | 0 | 2 | 2 | 0 | 5 | 0 | 0 | 9 | | |
| | | | Afr. | M. | 0 | 0 | 0 | 0 | 0 | 0 | 0 | 0 | 0 | | |
| | | | | F. | 0 | 1 | 0 | 2 | 0 | 1 | 1 | 0 | 5 | | |
| 176 | Vagina | Ca. | Eur. | M. | 0 | 0 | 0 | 0 | 0 | 0 | 0 | 0 | 0 | 30 | |
| | | | | F. | 0 | 0 | 0 | 0 | 2 | 2 | 1 | 1 | 6 | | |
| | | | Afr. | M. | 0 | 0 | 0 | 0 | 0 | 0 | 0 | 0 | 0 | | |
| | | | | F. | 0 | 1 | 4 | 3 | 7 | 4 | 5 | 0 | 24 | | |
| 177 | Prostate | Ca. | Eur. | M. | 0 | 0 | 0 | 0 | 0 | 6 | 4 | 0 | 10 | 20 | + 2 |
| | | | | F. | 0 | 0 | 0 | 0 | 0 | 0 | 0 | 0 | 0 | | |
| | | | Afr. | M. | 0 | 0 | 0 | 1 | 1 | 2 | 6 | 0 | 10 | | |
| | | | | F. | 0 | 0 | 0 | 0 | 0 | 0 | 0 | 0 | 0 | | |
| 178 | Testes | Various neoplasms | Eur. | M. | 0 | 0 | 1 | 1 | 1 | 1 | 0 | 0 | 4 | 5 | |
| | | | | F. | 0 | 0 | 0 | 0 | 0 | 0 | 0 | 0 | 0 | | |
| | | | Afr. | M. | 0 | 0 | 0 | 1 | 0 | 0 | 0 | 0 | 1 | | |
| | | | | F. | 0 | 0 | 0 | 0 | 0 | 0 | 0 | 0 | 0 | | |
| 179A | Scrotum | Ca. | Eur. | M. | 0 | 0 | 0 | 1 | 0 | 0 | 0 | 0 | 1 | 3 | |
| | | | | F. | 0 | 0 | 0 | 0 | 0 | 0 | 0 | 0 | 0 | | |
| | | | Afr. | M. | 0 | 0 | 0 | 1 | 0 | 1 | 0 | 0 | 2 | | |
| | | | | F. | 0 | 0 | 0 | 0 | 0 | 0 | 0 | 0 | 0 | | |
| 179B | Penis | Ca. | Eur. | M. | 0 | 0 | 0 | 0 | 0 | 1 | 1 | 0 | 2 | 31 | + 4 |
| | | | | F. | 0 | 0 | 0 | 0 | 0 | 0 | 0 | 0 | 0 | | |
| | | | Afr. | M. | 0 | 1 | 1 | 5 | 12 | 5 | 3 | 2 | 29 | | |
| | | | | F. | 0 | 0 | 0 | 0 | 0 | 0 | 0 | 0 | 0 | | |
| 180 | Kidney | Various neoplasms | Eur. | M. | 0 | 0 | 0 | 0 | 1 | 2 | 0 | 0 | 3 | 12 | + 2 |
| | | | | F. | 0 | 0 | 0 | 0 | 1 | 1 | 0 | 0 | 2 | | |
| | | | Afr. | M. | 1 | 1 | 1 | 1 | 0 | 0 | 0 | 0 | 4 | | |
| | | | | F. | 1 | 0 | 0 | 2 | 0 | 0 | 0 | 0 | 3 | | |
| 181A | Bladder | Ca. | Eur. | M. | 0 | 0 | 0 | 0 | 1 | 0 | 2 | 0 | 3 | 103 | + 7 |
| | | | | F. | 0 | 0 | 0 | 1 | 0 | 0 | 1 | 0 | 2 | | |
| | | | Afr. | M. | 0 | 0 | 8 | 16 | 23 | 9 | 9 | 2 | 67 | | |
| | | | | F. | 0 | 0 | 2 | 8 | 8 | 6 | 6 | 1 | 31 | | |
| 190 | Skin | Melanoblastoma | Eur. | M. | 0 | 0 | 0 | 0 | 0 | 0 | 0 | 0 | 0 | 28 | |
| | | | | F. | 0 | 0 | 0 | 0 | 1 | 0 | 1 | 0 | 2 | | |
| | | | Afr. | M. | 0 | 0 | 2 | 1 | 7 | 2 | 3 | 1 | 16 | | |
| | | | | F. | 0 | 0 | 1 | 2 | 1 | 0 | 4 | 2 | 10 | | |
| 191A 191B | ,, | Basal cell ca. Other ca. | Eur. | M. | 0 | 0 | 3 | 5 | 20 | 23 | 32 | 6 | 89 | 256 | + 9 |
| | | | | F. | 0 | 0 | 0 | 2 | 6 | 13 | 15 | 3 | 39 | | |
| | | | Afr. | M. | 1 | 6 | 12 | 15 | 26 | 12 | 4 | 3 | 79 | | |
| | | | | F. | 0 | 2 | 7 | 9 | 15 | 3 | 13 | 0 | 49 | | |
| 191D | ,, | Kaposi's tumour | Eur. | M. | 0 | 0 | 0 | 2 | 0 | 0 | 0 | 0 | 2 | 26 | |
| | | | | F. | 0 | 0 | 0 | 0 | 0 | 1 | 0 | 0 | 1 | | |
| | | | Afr. | M. | 0 | 2 | 2 | 3 | 5 | 7 | 2 | 1 | 22 | | |
| | | | | F. | 0 | 0 | 0 | 0 | 0 | 0 | 1 | 0 | 1 | | |
| 192A | Eye | Glioma | Eur. | M. | 0 | 0 | 0 | 0 | 0 | 0 | 0 | 0 | 0 | 7 | |
| | | | | F. | 1 | 0 | 0 | 0 | 0 | 0 | 1 | 0 | 2 | | |
| | | | Afr. | M. | 3 | 0 | 0 | 0 | 0 | 0 | 0 | 0 | 3 | | |
| | | | | F. | 2 | 0 | 0 | 0 | 0 | 0 | 0 | 0 | 2 | | |

TABLE II—*cont.*

| No. Int. Nom. | Site | Type | Race | Sex | Age groups 1–10 | 10–20 | 20–30 | 30–40 | 40–50 | 50 60 | Over 60 | ? | Total | Grand Total | Coloured |
|---|---|---|---|---|---|---|---|---|---|---|---|---|---|---|---|
| 192C | Eye | Various neoplasms | Eur. | M. | 0 | 1 | 0 | 0 | 1 | 0 | 0 | 1 | 3 | | |
| | | | | F. | 0 | 0 | 0 | 0 | 0 | 0 | 0 | 0 | 0 | | |
| | | | Afr. | M. | 1 | 2 | 3 | 5 | 2 | 1 | 0 | 0 | 14 | 34 | + 1 |
| | | | | F. | 3 | 0 | 3 | 4 | 4 | 1 | 2 | 0 | 17 | | |
| 193A | Brain | Glioma | Eur. | M. | 2 | 0 | 0 | 0 | 2 | 1 | 1 | 2 | 8 | | |
| | | | | F. | 0 | 0 | 0 | 0 | 0 | 0 | 0 | 0 | 0 | | |
| | | | Afr. | M. | 2 | 4 | 3 | 3 | 2 | 0 | 0 | 0 | 14 | 24 | |
| | | | | F. | 1 | 0 | 0 | 0 | 1 | 0 | 0 | 0 | 2 | | |
| 194 | Thyroid gland | Ca. | Eur. | M. | 0 | 0 | 1 | 0 | 0 | 0 | 0 | 0 | 1 | | |
| | | | | F. | 0 | 0 | 0 | 0 | 1 | 0 | 0 | 0 | 1 | | |
| | | | Afr. | M. | 0 | 1 | 2 | 0 | 0 | 0 | 0 | 0 | 3 | 11 | + 1 |
| | | | | F. | 0 | 0 | 2 | 1 | 2 | 1 | 0 | 0 | 6 | | |
| 196 | Jawbone | Various neoplasms | Eur. | M. | 0 | 0 | 2 | 0 | 1 | 0 | 0 | 0 | 3 | | |
| | | | | F. | 0 | 0 | 0 | 0 | 0 | 0 | 0 | 1 | 1 | | |
| | | | Afr. | M. | 0 | 6 | 1 | 3 | 1 | 0 | 1 | 0 | 12 | 24 | |
| | | | | F. | 0 | 1 | 2 | 4 | 1 | 0 | 0 | 0 | 8 | | |
| 196C | Other bones | Sarcoma | Eur. | M. | 0 | 1 | 0 | 0 | 0 | 1 | 0 | 0 | 2 | | |
| | | | | F. | 0 | 0 | 0 | 0 | 0 | 0 | 0 | 0 | 0 | | |
| | | | Afr. | M. | 1 | 2 | 3 | 2 | 0 | 0 | 0 | 0 | 8 | 13 | + 2 |
| | | | | F. | 0 | 0 | 1 | 1 | 0 | 0 | 1 | 0 | 3 | | |
| 197 | Connective tissue | Sarcoma | Eur. | M. | 1 | 1 | 1 | 3 | 2 | 1 | 0 | 1 | 10 | | |
| | | | | F. | 0 | 0 | 2 | 4 | 3 | 1 | 3 | 0 | 13 | | |
| | | | Afr. | M. | 3 | 7 | 9 | 9 | 9 | 4 | 2 | 3 | 46 | 84 | + 6 |
| | | | | F. | 2 | 2 | 2 | 4 | 3 | 2 | 0 | 0 | 15 | | |
| 198 | Lymph nodes | Secondary: Ca. Sarcoma | Eur. | M. | 0 | 0 | 0 | 1 | 1 | 2 | 5 | 1 | 10 | | |
| | | | | F. | 0 | 0 | 0 | 1 | 0 | 1 | 1 | 0 | 3 | | |
| | | | Afr. | M. | 0 | 2 | 5 | 1 | 2 | 2 | 2 | 1 | 15 | 31 | + 2 |
| | | | | F. | 0 | 0 | 0 | 1 | 1 | 0 | 1 | 0 | 3 | | |
| 200.0 | Various sites | Reticulum cell sarcoma | Eur. | M. | 0 | 0 | 0 | 0 | 0 | 1 | 1 | 0 | 2 | | |
| | | | | F. | 1 | 0 | 0 | 2 | 0 | 0 | 0 | 0 | 3 | | |
| | | | Afr. | M. | 3 | 0 | 0 | 0 | 1 | 0 | 0 | 1 | 5 | 14 | + 2 |
| | | | | F. | 3 | 0 | 1 | 0 | 0 | 0 | 0 | 0 | 4 | | |
| 200.1 | Ditto | Lympho-sarcoma | Eur. | M. | 2 | 0 | 0 | 1 | 2 | 1 | 3 | 0 | 9 | | |
| | | | | F. | 0 | 0 | 0 | 0 | 0 | 0 | 0 | 0 | 0 | | |
| | | | Afr. | M. | 10 | 4 | 3 | 4 | 3 | 2 | 0 | 0 | 26 | 52 | + 3 |
| | | | | F. | 6 | 3 | 2 | 2 | 1 | 1 | 2 | 0 | 17 | | |
| 201 | Lymph nodes | Hodgkin's disease | Eur. | M. | 0 | 1 | 4 | 1 | 3 | 1 | 0 | 2 | 12 | | |
| | | | | F. | 0 | 1 | 3 | 1 | 0 | 0 | 1 | 0 | 6 | | |
| | | | Afr. | M. | 0 | 7 | 9 | 9 | 6 | 2 | 1 | 0 | 34 | 60 | + 6 |
| | | | | F. | 0 | 1 | 1 | 1 | 2 | 2 | 1 | 0 | 8 | | |
| 203 204B 204E | Various sites | Myeloma Lymphatic and myeloid leukaemia | Eur. | M. | 0 | 0 | 0 | 1 | 0 | 0 | 0 | 0 | 1 | | |
| | | | | F. | 0 | 0 | 0 | 0 | 0 | 0 | 0 | 0 | 0 | | |
| | | | Afr. | M. | 1 | 1 | 0 | 5 | 1 | 0 | 0 | 0 | 8 | 13 | |
| | | | | F. | 0 | 0 | 0 | 1 | 1 | 1 | 1 | 0 | 4 | | |
| | | Totals | Eur. | M. | 5 | 6 | 13 | 20 | 57 | 76 | 106 | 20 | 303 | | |
| | | | | F. | 2 | 3 | 12 | 48 | 68 | 76 | 56 | 12 | 277 | | |
| | | | Afr. | M. | 28 | 115 | 182 | 176 | 174 | 87 | 61 | 19 | 842 | | |
| | | | | F. | 19 | 18 | 67 | 92 | 99 | 60 | 65 | 11 | 431 | | |

Eur.—European.
Afr.—African.

TABLE III.—*Distribution by Site, Type and Sex, of all Malignant Neoplasms in Europeans, Africans and Coloureds* from April* 1944–*December* 31, 1957

| No. Int. Nom. | Site | Type | European M. | % | European F. | % | African M. | % | African F. | % | Coloured M. | Coloured F. | Total | Percentage of all neoplasms |
|---|---|---|---|---|---|---|---|---|---|---|---|---|---|---|
| 140 | Lip | Ca. | 8 | 2·64 | 0 | 0 | 3 | 0·36 | 7 | 1·62 | 0 | 0 | 18 | 0·91 |
| 141 | Tongue | ,, | 5 | 1·65 | 0 | 0 | 4 | 0·47 | 0 | 0 | 1 | 0 | 11 | 0·56 |
| | | Sarcoma | 0 | 0 | 0 | 0 | 0 | 0 | 0 | 0 | 0 | 1 | | |
| 142.1 | Salivary gland parotid | Ca. | 1 | 0·33 | 0 | 0 | 0 | 0 | 5 | 1·16 | 1 | 0 | 8 | 0·40 |
| | | Sarcoma | 0 | 0 | 0 | 0 | 1 | 0·12 | 0 | 0 | 0 | 0 | | |
| 144 | Mouth | Ca. | 4 | 1·32 | 1 | 0·36 | 12 | 1·54 | 8 | 2·1 | 1 | 0 | 28 | 1·42 |
| | | Sarcoma | 0 | 0 | 0 | 0 | 1 | | 1 | | 0 | 0 | | |
| 145A | Tonsil | Ca. | 3 | 0·99 | 0 | 0 | 3 | 0·36 | 0 | 0 | 2 | 0 | 8 | 0·40 |
| 146 | Nasopharynx | ,, | 2 | 0·66 | 1 | 0·36 | 0 | 0 | 0 | 0 | 0 | 0 | 3 | 0·15 |
| 150 | Oesophagus | ,, | 1 | 0·33 | 1 | 0·36 | 4 | 0·47 | 0 | 0 | 2 | 0 | 8 | 0·40 |
| 151 | Stomach | ,, | 27 | 8·91 | 13 | 4·7 | 9 | 1·06 | 0 | 0 | 2 | 1 | 52 | 2·63 |
| 152C | Small instestine | ,, | 2 | 0·66 | 3 | 1·08 | 1 | 0·12 | 1 | 0·23 | 0 | 0 | 7 | 0·35 |
| 153A | Large intestine | ,, | 15 | 5·61 | 5 | 2·17 | 3 | 0·36 | 0 | 0 | 3 | 1 | 31 | 1·57 |
| | | Carcinoid | 2 | | 1 | | 0 | 0 | 0 | 0 | 0 | 1 | | |
| 154 | Rectum | Ca. | 10 | 3·31 | 2 | 0·72 | 1 | 0·12 | 3 | 0·70 | 1 | 3 | 20 | 1·02 |
| 155A | Liver | Ca. (Primary) | 8 | 2·64 | 3 | 1·09 | 363 | 43·11 | 63 | 14·62 | 5 | 1 | 443 | 22·42 |
| 155C | Gall bladder | Ca. | 4 | 1·32 | 7 | 2·51 | 1 | 0·12 | 0 | 0 | 1 | 1 | 14 | 0·70 |
| 157 | Pancreas | ,, | 3 | 0·99 | 0 | 0 | 8 | 0·95 | 1 | 0·23 | 1 | 1 | 14 | 0·70 |
| 158 | Abdominal cavity | ,, | 2 | 0·66 | 3 | 1·09 | 3 | 0·36 | 3 | 0·70 | 0 | 1 | 12 | 0·61 |
| 160A | Nose | ,, | 2 | 0·66 | 0 | 0 | 1 | 0·12 | 0 | 0 | 0 | 0 | 3 | 0·15 |
| 161 | Larynx | ,, | 15 | 4·95 | 1 | 0·36 | 2 | 0·24 | 0 | 0 | 2 | 0 | 20 | 1·02 |
| 162C | Lung Bronchus | ,, | 14 | 4·62 | 1 | 0·36 | 3 | 0·36 | 0 | 0 | 1 | 1 | 20 | 1·02 |
| 170 | Breast | ,, | 0 | 0 | 66 | 23·83 | 1 | 0·12 | 32 | 7·42 | 0 | 12 | 111 | 5·61 |
| 171 | Cervix uteri | ,, | 0 | 0 | 55 | 19·87 | 0 | 0 | 89 | 20·65 | 0 | 21 | 165 | 8·36 |
| 172 | Corpus uteri | ,, | 0 | 0 | 13 | 5·05 | 0 | 0 | 4 | 0·93 | 0 | 3 | 22 | 1·11 |
| | | Sarc. | 0 | 0 | 1 | | 0 | 0 | 0 | | 0 | 1 | | |
| 173 | Uterus | Chorion-epithelioma | 0 | 0 | 10 | 3·6 | 0 | 0 | 2 | 0·46 | 0 | 4 | 16 | 0·81 |
| 175A | Ovary | Ca. | 0 | 0 | 9 | 3·25 | 0 | 0 | 5 | 1·16 | 0 | 0 | 14 | 0·70 |
| 176 | Vagina | ,, | 0 | 0 | 6 | 2·17 | 0 | 0 | 24 | 5·57 | 0 | 0 | 30 | 1·52 |
| 177 | Prostate | ,, | 10 | 3·31 | 0 | 0 | 10 | 1·2 | 0 | 0 | 2 | 0 | 22 | 1·11 |
| 178 | Testes | Seminoma | 2 | 1·32 | 0 | 0 | 1 | 0·12 | 0 | 0 | 0 | 0 | 5 | 0·25 |
| | | Sarcoma | 2 | | 0 | 0 | 0 | 0 | 0 | 0 | 0 | 0 | | |
| 179A | Scrotum | Ca. | 1 | 0·33 | 0 | 0 | 2 | 0·24 | 0 | 0 | 0 | 0 | 3 | 0·15 |
| 179B | Penis | ,, | 2 | 0·66 | 0 | 0 | 29 | 3·44 | 0 | 0 | 4 | 0 | 35 | 1·77 |
| 180 | Kidney | Various neoplasms | 3 | 0·99 | 2 | 0·72 | 4 | 0·47 | 3 | 0·70 | 0 | 2 | 14 | 0·70 |
| 181A | Bladder | Ca. | 3 | 0·99 | 2 | 0·72 | 67 | 7·96 | 31 | 7·19 | 7 | 0 | 110 | 5·56 |
| 190 | Skin | Melano-blastoma | 0 | 0 | 2 | 0·72 | 16 | 1·9 | 10 | 2·3 | 0 | 0 | 28 | 1·42 |
| 191A | ,, | Basal cell Ca. | 73 | 24·08 | 32 | 11·55 | 14 | 1·66 | 15 | 3·48 | 5 | 1 | 140 | 7·09 |
| 191D | ,, | Other ca. | 16 | 5·27 | 7 | 2·51 | 65 | 7·72 | 34 | 7·89 | 2 | 1 | 125 | 6·33 |
| 191D | ,, | Kaposi's tumour | 2 | 0·66 | 1 | 0·36 | 22 | 2·61 | 1 | 0·23 | 0 | 0 | 26 | 1·32 |
| 192A | Eye | Glioma | 0 | 0 | 2 | 0·72 | 3 | 0·36 | 2 | 0·46 | 0 | 0 | 7 | 0·35 |
| 192C | ,, | Various neoplasms | 3 | 0·99 | 0 | 0 | 14 | 1·66 | 17 | 3·94 | 0 | 1 | 35 | 1·77 |
| 193A | Brain | Ditto | 8 | 2·64 | 0 | 0 | 14 | 1·66 | 2 | 0·46 | 0 | 0 | 24 | 1·21 |
| 194 | Thyroid gland | Ca. | 1 | 0·33 | 1 | 0·36 | 3 | 0·36 | 6 | 1·4 | 1 | 0 | 12 | 0·61 |
| 196 | Jawbone | Adamantinoma | 1 | 0·99 | 0 | 0·36 | 5 | 1·43 | 4 | 1·86 | 0 | 0 | 24 | 1·21 |
| | | Sarcoma | 2 | | 1 | | 7 | | 4 | | 0 | 0 | | |
| 196C | Other bones | Sarcoma | 2 | 0·66 | 0 | 0 | 8 | 0·95 | 3 | 0·70 | 2 | 0 | 15 | 0·76 |
| 197 | Connective tissue | ,. | 10 | 3·31 | 13 | 4·7 | 46 | 5·46 | 15 | 3·48 | 2 | 4 | 90 | 4·55 |

TABLE III—*cont.*

| No. Int. Nom. | Site | Type | European M. | % | F. | % | African M. | % | F. | % | Coloured M. | F. | Total | Percentage of all neoplasms |
|---|---|---|---|---|---|---|---|---|---|---|---|---|---|---|
| 198 | Lymph nodes | Secondary ca.<br>Sarcoma | 9<br>1 | 3·31 | 2<br>1 | 1·09 | 14<br>1 | 1·78 | 3<br>0 | 0·70<br>0 | 1<br>0 | 1<br>0 | 33 | 1·67 |
| 200.0 | Various sites | Reticulum cell sarcoma | 2 | 0·66 | 3 | 1·09 | 5 | 0·59 | 4 | 0·93 | 1 | 1 | 16 | 0·81 |
| 200.1 | Ditto | Lympho-sarcoma | 9 | 2·97 | 0 | 0 | 26 | 3·08 | 17 | 3·94 | 3 | 0 | 55 | 2·78 |
| 201 | Lymph nodes | Hodgkin's disease | 12 | 3·95 | 6 | 2·17 | 34 | 4·04 | 8 | 1·86 | 3 | 3 | 66 | 3·34 |
| 203 | Bones | Myeloma | 1 | 0·33 | 0 | 0 | 2 | 0·24 | 1 | 0·23 | 0 | 0 | 4 | 0·20 |
| 204B | Blood | Lymphatic leukaemia | 0 | 0 | 0 | 0 | 0 | 0 | 3 | 0·70 | 1 | 0 | 4 | 0·20 |
| 204A | ,, | Myeloid leukaemia | 0 | 0 | 0 | 0 | 6 | 0·71 | 0 | 0 | 0 | 0 | 6 | 0·30 |
| | | Totals | 303 | 100 | 277 | 100 | 842 | 100 | 431 | 100 | 57 | 67 | 1977 | 100 |

* As a total of only 124 neoplasms were observed amongst Coloured peoples, the percentage frequency of these tumours has not been included.

From this analysis, several trends in the tumour ratio frequency can be recognized in Lourenço Marques, namely,

1. A predilection of cancer for the liver in African males and females as compared with the European ;
2. carcinoma of the cervix uteri is relatively more frequent than carcinoma of the corpus uteri in African as compared with European women ;
3. breast carcinoma appears to be less common in African than in European women ;
4. skin cancer in the European male is more common than in the African male although the types of skin tumours developed by the two races are not the same ;
5. carcinoma of the lung, oesophagus, stomach, large bowel and rectum are relatively uncommon in the African as compared with the European (Table II).

Data for the Indian, Chinese and Coloured groups are too limited for analysis and are merely included in Table II as additional information on tumour frequency.

Preliminary information from our rates survey discloses that only a small percentage of Africans live beyond the age of 65 years. The age structure of the population may influence profoundly the site incidence of cancer. Nevertheless, one fact is outstanding, namely, that as far as liver cancer is concerned, it occurs with unusual frequency under the age of 40 years (Table VI). Of a total of 363 cases of primary carcinoma of the liver in males, no less than 182 occurred under the age of 30 years while in females, 29 of 63 were present under the age of 30 years. It may well be that when corrected for the age distribution of the population, carcinoma of the liver may increase still further with age. But, at present, the early development of liver carcinoma in young people cannot be disputed.

TABLE IV.—*Primary Carcinoma of the Liver, by Race, Sex and Age*

| No. Int. Nom. | Site | Type | Race | Sex | Age groups 1–10 | 10–20 | 20–30 | 30–40 | 40–50 | 50–60 | Over 60 | ? | Total |
|---|---|---|---|---|---|---|---|---|---|---|---|---|---|
| 155a | Liver | Carcinoma (Primary) | European | M. | 0 | 0 | 0 | 0 | 4 | 1 | 3 | 0 | 8 |
| | | | | F. | 0 | 0 | 0 | 0 | 0 | 2 | 1 | 0 | 3 |
| | | | African | M. | 2 | 67 | 113 | 83 | 53 | 26 | 15 | 4 | 363 |
| | | | | F. | 1 | 7 | 21 | 16 | 7 | 5 | 5 | 1 | 63 |
| | | | Coloured | M. | 1 | 0 | 0 | 2 | 0 | 1 | 1 | 0 | 5 |
| | | | | F. | 0 | 1 | 0 | 0 | 0 | 0 | 0 | 0 | 1 |
| | | | Total | | 4 | 75 | 134 | 101 | 64 | 35 | 25 | 5 | 443 |

*The frequency ratio of malignant neoplasms in Africans since the establishment of the Cancer Survey Unit (Table VII)*

As already mentioned above, a special cancer survey was initiated in May 1956. As a result, interest in the cancer problem grew rapidly amongst the doctors in the city of Lourenço Marques and in the hospital. This did not affect to any extent the number of specimens sent to the laboratory from outlying regions but it did increase the number of histological examinations of material obtained at operation in the hospital. The number of autopsies increased to over 80 per cent of all patients dying in the hospital.

During the past 19 months, 210 malignant neoplasms found in Africans were diagnosed histologically. In the male African, the liver accounted for 45 per cent, the skin for 11·4 per cent and the urinary bladder for 8·2 per cent of all cancers. That is to say, 64·6 per cent of all tumours occurred in these three sites. In females, 18·1 per cent of carcinomata were observed in the cervix uteri, 11·3 per cent in the liver, 11·36 per cent in the bladder and 11·5 per cent in the skin. These tumours represented 52 per cent of all neoplasms in African women. Up to the present, 16 carcinomata of the cervix uteri have been detected and not a single carcinoma of the body of the uterus. Carcinoma of the oesophagus and of the lung and bronchus were uncommon. Only 2 cases occurred in 210 malignant growths in both sexes. Sarcoma of the jaw and other regions of the skeleton yielded 9·8 per cent of tumours, while primary malignant neoplasms of the lymphoid tissues were found in 9·0 per cent of cases. The frequency of carcinoma of the pancreas remained low (0·82 per cent).

A comparison of the data collected over the 19-month period with that accumulated over the entire previous 13 years reveals that while the frequency ratios were not always identical, the general trend in site incidence remained the same. A relatively small series of 210 neoplasms collected over a period of 19 months, therefore, can yield useful suggestive information about the frequency ratio of the more commonly occurring tumours.

*Cancer rates in the population of Lourenço Marques*

The cancer rates survey was also initiated 19 months ago and relates to the African population numbering approximately 100,000. One hundred and sixty-one tumours have been studied. As previously mentioned, unless confirmed histologically, all malignant cases diagnosed radiologically and clinically were excluded from the above analysis.

If the maternity cases are also excluded, four times as many males were admitted to hospital as females, indicating that some sociological factor may have determined the male preference for seeking medical attention and admission to

TABLE V.—*Ratio Survey of Malignant Neoplasms in Africans of Lourenço Marques from May* 1956–*December* 1957

| No. Int. Nom. | Site | Type | Male: Actual number | Male: % | Female: Actual number | Female: % | Total M. and F. | Percentage M. and F. |
|---|---|---|---|---|---|---|---|---|
| 141 | Tongue | Ca. | 1 | 0·82 | 0 | 0 | 1 | 0·48 |
| 142.1 | Salivary gland | ,, | 0 | 0 | 1 | 1·14 | 1 | 0·48 |
| 144 | Mouth | ,, | 1 | 0·82 | 1 | 1·14 | 2 | 0·95 |
| 153A | Large intestine | ,, | 1 | 0·82 | 0 | 0 | 1 | 0·48 |
| 155A | Liver | ,, | 55 | 45·06 | 10 | 11·36 | 65 | 30·96 |
| 157 | Pancreas | ,, | 1 | 0·82 | 0 | 0 | 1 | 0·48 |
| 158 | Abdominal cavity | ,, | 0 | 0 | 2 | 2·27 | 2 | 0·95 |
| 161 | Larynx | ,, | 2 | 1·64 | 0 | 0 | 2 | 0·95 |
| 162C | Lung and bronchus | ,, | 2 | 1·64 | 0 | 0 | 2 | 0·95 |
| 170 | Breast | ,, | 0 | 0 | 10 | 11·36 | 10 | 4·77 |
| 171 | Cervix uteri | ,, | 0 | 0 | 16 | 18·18 | 16 | 7·62 |
| 175A | Ovary | Sarcoma | 0 | 0 | 1 | 1·14 | 1 | 0·48 |
| 176 | Vagina | Ca. | 0 | 0 | 3 | 3·41 | 3 | 1·43 |
| 177 | Prostate | ,, | 1 | 0·82 | 0 | 0 | 1 | 0·48 |
| 179B | Penis | ,, | 1 | 0·82 | 0 | 0 | 1 | 0·48 |
| 180 | Kidney | ,, | 1 | 0·82 | 0 | 0 | 2 (both rows) | 0·95 (both rows) |
| | | Sarcoma | 0 | 0 | 1 | 1·14 | | |
| 181 | Bladder | Ca. | 10 | 8·2 | 10 | 11·36 | 20 | 9·52 |
| 190 | Skin | Malignant melanoma | 1 | 0·82 | 1 | 1·14 | 2 | 0·95 |
| 191A | ,, | Basal cell ca. | 4 | 3·28 | 3 | 3·41 | 7 | 3·33 |
| 191B | ,, | Miscellaneous ca. | 6 | 4·92 | 6 | 6·80 | 12 | 5·71 |
| 191D | ,, | Kaposis | 3 | 2·40 | 0 | 0 | 3 | 1·43 |
| 192C | Eye | Ca. | 1 | 0·82 | 2 | 2·27 | 5 (both rows) | 2·38 (both rows) |
| | | Sarcoma | 1 | 0·82 | 1 | 1·14 | | |
| 193A | Brain | Glioma | 3 | 2·46 | 0 | 0 | 3 | 1·43 |
| 194 | Thyroid gland | Ca. | 0 | 0 | 2 | 2·27 | 2 | 0·95 |
| 196 | Jawbone | Sarcoma | 8 | 6·56 | 7 | 7·95 | 15 | 7·14 |
| 196C | Other bones | ,, | 4 | 3·28 | 1 | 1·14 | 5 | 2·38 |
| 197 | Connective tissue | ,, | 1 | 0·82 | 3 | 3·41 | 4 | 1·90 |
| 198 | Lymph nodes | Secondary ca. | 2 | 1·64 | 0 | 0 | 2 | 0·95 |
| 200.0 | Various sites | Reticulo-endothelial sarcoma | 2 | 1·64 | 2 | 2·27 | 4 | 1·90 |
| 200.1 | ,, | Lympho-sarcoma | 4 | 3·28 | 1 | 1·14 | 5 | 2·38 |
| 201 | Lymph nodes | Hodgkin's disease | 3 | 2·46 | 1 | 1·14 | 4 | 1·9 |
| 203 | Bones | Myeloma | 0 | 0 | 1 | 1·14 | 1 | 0·48 |
| 204B | Blood | Lymphatic leukaemia | 1 | 0·82 | 1 | 1·14 | 2 | 0·95 |
| 204E | ,, | Myeloid leukaemia | 2 | 1·64 | 1 | 1·14 | 3 | 1·43 |
| | | Total | 122 | 100 | 88 | 100 | 210 | 100 |

hospital. While the data collected during the first 19 months of our rates survey in a relatively small population can only afford an indication of the trends to be expected, nevertheless, even at this early stage, it is evident that the frequency of even the common tumours is further emphasized (Table VI). Thus, of 110 malignant neoplasms in males, 71 (64·54 per cent) were primary in the liver and of 51 malignant neoplasms in females, 13 (25·5 per cent) were primary in the liver. The bladder accounted for 8·18 per cent of tumours, and the skin for 7·27 per cent in males, these sites together with the liver accounting for 71·81 per cent of all malignant neoplasms. Again, the low frequency of lung carcinoma is noteworthy. However, almost one-third of all cancers in women were primary in the cervix uteri.

In general, the first 19 months of the intensive rates survey confirmed the trend observed in the cancer frequency ratios in all the material obtained in the past 13 years. As the survey progresses, it may well be that the frequency ratio of liver cancer will decline a little but, at present, there can be little doubt that amongst males, the liver is still the most frequent site of cancer. In females

TABLE VI.—*Rates Survey of Malignant Neoplasms in Africans of Lourenço Marques from May* 1956–*December* 1957

| No. Int. Nom. | Site | Type | Male Actual number | Male % | Female Actual number | Female % | Total M. and F. | Percentage M. and F. |
|---|---|---|---|---|---|---|---|---|
| 142.1 | Salivary gland Parotid | Ca. | 0 | 0 | 1 | 1·96 | 1 | 0·62 |
| 144 | Mouth | ,, | 1 | 0·91 | 1 | 1·96 | 2 | 1·24 |
| 151 | Stomach | ,, | 1 | 0·91 | 0 | 0 | 1 | 0·62 |
| 153A | Large intestine | ,, | 1 | 0·91 | 0 | 0 | 1 | 0·62 |
| 155A | Liver | ,, | 71 | 64·54 | 13 | 25·5 | 84 | 52·17 |
| 146 | Nasopharynx | ,, | 1 | 0·91 | 0 | 0 | 1 | 0·62 |
| 170 | Breast | ,, | 0 | 0 | 1 | 1·96 | 1 | 0·62 |
| 171 | Cervix uteri | ,, | 0 | 0 | 15 | 29·42 | 15 | 9·31 |
| 176 | Vulva | ,, | 0 | 0 | 1 | 1·96 | 1 | 0·62 |
| 177 | Prostate | ,, | 4 | 3·63 | 0 | 0 | 4 | 2·48 |
| 179B | Penis | ,, | 3 | 2·73 | 0 | 0 | 3 | 1·85 |
| 180 | Kidney | Various neoplasms | 1 | 0·91 | 1 | 1·96 | 2 | 1·24 |
| 181 | Bladder | Ca. | 9 | 8·18 | 4 | 7·84 | 13 | 8·07 |
| 190 | Skin | Melanoblastoma | 2 | 1·82 | 0 | 0 | 2 | 1·24 |
| 191A,B | ,, | Ca. | 5 | 4·54 | 2 | 3·92 | 7 | 4·44 |
| 191D | ,, | Kaposi | 1 | 0·91 | 0 | 0 | 1 | 0·62 |
| 192C | Eye | Ca. | 1 | 0·91 | 1 | } 3·92 | } 3 | } 1·85 |
| | | Sarcoma | 0 | 0 | 1 | | | |
| 193A | Brain | Glioma | 3 | 2·73 | 2 | 3·92 | 5 | 3·10 |
| 194 | Thyroid gland | Ca. | 0 | 0 | 2 | 3·92 | 2 | 1·24 |
| 196 | Jawbone | Sarcoma | 1 | 0·91 | 0 | 0 | } 2 | } 1·24 |
| | | Adamantinoma | 1 | 0·91 | 0 | 0 | | |
| 197 | Connective tissue | Sarcoma | 1 | 0·91 | 2 | 3·92 | 3 | 1·85 |
| 200.1 | Various sites | Lymphosarcoma | 1 | 0·91 | 1 | 1·96 | 2 | 1·24 |
| 201 | Lymph nodes | Hodgkin's disease | 0 | 0 | 1 | 1·96 | 1 | 0·62 |
| 204A | Blood | Myeloid leukaemia | 2 | 1·82 | 2 | 3·92 | 4 | 2·48 |
| | | Total | 110 | 100 | 51 | 100 | 161 | 100 |

carcinoma of the cervix occurs more frequently than all other cancers, with cancer of the liver ranking next in order of importance and this despite the smaller number of women seeking hospital treatment. Both in males and females, cancer of the bladder is equally common, being second in order of frequency in males and third in the females. It is hoped that when the cancer rates survey is completed, it will be possible to know, with greater confidence whether carcinoma of the stomach, oesophagus, breast and lung are indeed lower in the African in Lourenço Marques, as the frequency ratio study seems to suggest. However, there is sufficient information to show that primary cancer of the liver is a particular problem in Lourenço Marques and justifies an intensive study into the aetological factors.

*Comparison of cancer frequency ratios in Mozambique with that in other parts of Africa*

With the exception of Kampala (1952–53) and Durban (1950–56), carcinoma of the liver in male Africans is the commonest tumour in all cancer surveys thus far reported from Johannesburg, Mozambique, Belgian Congo and Nigeria and reaches the highest frequency ratio in Lourenço Marques where liver carcinoma is twice as common as in Johannesburg and three times as common as in Kampala. It is of interest to note that Findlay (1940–45) reported 60 tumours in West African soldiers aged 18 to 40 years and of these 37 were primary cancer of the liver. Moreover, calculations made from the data presented by Elmes and Baldwin (1947) for Nigeria reveal a frequency ratio of 11·9 per cent for primary carcinoma of the liver.

The frequency ratio of carcinoma of the skin ranges from 11·2 to 16·3 per cent and is the commonest cancer in Kampala and Durban, second in order of frequency in Mozambique and Johannesburg (Higginson and Oettlé, 1958, personal communication) and third in the Stanleyville and in the data submitted by Berman (1935) for Johannesburg. Tumours of the bladder constitute between 4 and 8 per cent of all neoplasms in Mozambique, Johannesburg and Kampala but less than 1 per cent in Stanleyville. In Mozambique, bladder tumours are almost twice as frequent as in Johannesburg and Kampala.

The relatively high frequency ratio of carcinoma of the lung and bronchus in Johannesburg (7·7 per cent) (Higginson and Oettlé, 1958, personal communication) and in Durban (7·7 per cent) contrasts with the lower frequency of tumours of this organ in Kampala (1·95 per cent) and the rarity of this tumour in Mozambique (less than 1 per cent) and in Dakar (Camain, 1954). Similarly, the high frequency ratio of carcinoma of the rectum in Johannesburg (8.0 per cent) differs from the lower frequency in Kampala (2·3 per cent) and the still lower frequency ratio (less than 1 per cent) in Mozambique and Stanleyville. Apparently, tumours of the gastro-intestinal tract are more common in Johannesburg and Kampala than in Mozambique and Stanleyville. The frequency of carcinoma of the penis appears to be considerably higher in Kampala (10·0 per cent) than in any other African territory (1–4 per cent).

*Females.*—The high frequency ratio of carcinoma of the cervix is a feature of all reports from Africa with the exception of Dakar. In Nigeria, tumours of the uterus ranked second in order of frequency.

Skin tumours were found first in order of frequency in Durban and second in Johannesburg (12·1 per cent) and Stanleyville (13·2 per cent) and third in Mozambique (13·9 per cent in both sexes). In Kampala, skin tumours constituted only

8 per cent of all tumours and only 3 per cent in the figures published by Berman (1935) for Johannesburg.

The frequency ratio of liver carcinoma in African females was only slightly higher (14·6 per cent) than that of skin carcinoma (13·8 per cent) in Mozambique and was at least twice as high as the frequency ratio of liver carcinoma in Johannesburg (Higginson and Oettlé (6·0 per cent), Berman (5·0 per cent), Stanleyville (4·5 per cent) and Kampala (4·1 per cent). Carcinoma of the liver was consistently lower amongst females than in males in all the surveys presented in Tables VII and VIII.

The frequency ratio of breast tumours was variable. Whereas breast carcinoma constituted 7·4 and 9·0 per cent of all tumours in Mozambique and Kampala respectively, the percentage frequency was 11·3 and 12·0 per cent in Johannesburg (Higginson and Oettlé) and Stanleyville (Thijs, 1957) and 25·3 and 21·4 per cent in Johannesburg (Berman, 1935) and Nigeria respectively. Squamous cell carcinoma of the vagina was consistently higher in Mozambique (5·5 per cent) than elsewhere while tumours of the ovary appeared to be unusually high in Stanleyville (8·8 per cent) and in Kampala (11.5 per cent). Carcinoma of the gastro-intestinal canal was unusual and involved mainly the stomach, the highest frequency ratio being reported in Johannesburg by Berman. Carcinoma of the rectum occurred in less than 1 per cent of tumours in all surveys, except in Kampala. Except in Durban (Wainwright and Roach, 1957) cancer of the lung and bronchus comprised 1·4 per cent or less of all tumours in females in all the surveys reported.

As in the African male, carcinoma of the bladder was high (7·9 per cent) in African females in Mozambique but was far less frequent in the other African territories. Camain (1954) from Dakar, reported on 1884 malignant neoplasms in Africans over an 11-year period. Unfortunately, he did not separate the sexes and therefore it is not possible to compare the Lourenço Marques tumours with those in Dakar. However, Camain does mention that two-thirds of the tumours were obtained from males and one-third from females. It would appear that the frequency ratio of primary liver cancer in Dakar is a little lower than in Lourenço Marques. Noteworthy in Dakar, as in Lourenço Marques, are the low frequency ratios of pancreatic, large bowel, lung and breast cancers. The ratio of cancer of the body to cancer of the cervix uteri in Dakar is as 1 : 5·7, which contrasts with the reported ratios from other parts of Africa.

From this comparative survey, whatever the limitations thereof, it is evident that in several territories south of the Sahara, primary liver cancer is the predominant malignant neoplasm in males, the only anomalous report being from Kampala. With the exception of Dakar and possibly also of Nigeria, carcinoma of the cervix uteri in females ranks first in importance amongst all neoplasms in African women. Both in males and in females, primary carcinoma of the liver, bladder and skin show a markedly higher frequency ratio in Mozambique than elsewhere in Africa. With the exception of Johannesburg and Kampala, carcinoma of the gastro-intestinal canal amongst Africans appears to be infrequent. At present, cancer of the lung and bronchus does not appear to be a problem of any significance amongst African females and, with the exception of Johannesburg, these tumours are also uncommon in African males.

The comparative analysis of the tumour frequency ratio reveals that primary liver cancer in males and cancer of the bladder in both sexes in Lourenço Marques

TABLE VII.—*Order of Frequency of Malignant Neoplasms in African Males in Different Parts of the Continent of Africa* ‡

**Lourenço Marques 1944–57 (Prates) — European†**

| Site | Total | % |
|---|---|---|
| Skin | 91 | 30·0 |
| Stomach | 27 | 8·9 |
| Colon | 17 | 5·6 |
| Larynx | 15 | 4·9 |
| Lung and bronchus | 14 | 4·6 |
| Hodgkin's disease | 12 | 3·9 |
| Sarcoma | 10 | 3·3 |
| Lymph nodes | 10 | 3·3 |
| Rectum | 10 | 3·3 |
| Prostate | 10 | 3·3 |
| Lip | 8 | 2·6 |
| Liver | 8 | 2·6 |
| Brain | 8 | 2·6 |
| Tongue | 5 | 1·6 |
| Bones | 5 | 1·6 |
| Mouth | 4 | 1·3 |
| Gall bladder | 4 | 1·3 |
| Testis | 4 | 1·3 |

**Lourenço Marques 1944–57 (Prates) — African**

| Site | Total | % |
|---|---|---|
| Liver | 363 | 43·1 |
| Skin | 117 | 13·9 |
| Lymphomata | 73 | 8·6 |
| Bladder | 67 | 7·9 |
| Sarcoma | 46 | 5·5 |
| Penis | 29 | 3·4 |
| Kaposi's tumour | 22 | 2·6 |
| Bones | 20 | 2·3 |
| Eye | 17 | 2·0 |
| Lymph nodes: (secondary) | 15 | 1·7 |
| Brain | 14 | 1·6 |
| Mouth | 13 | 1·5 |
| Prostate | 10 | 1·2 |
| Stomach | 9 | 1·0 |

**Johannesburg 1926–33 (Berman)**

| Site | Total | % |
|---|---|---|
| Liver | 36 | 31·0 |
| Stomach | 17 | 14·7 |
| Skin | 13 | 11·2 |
| Bladder | 5 | 4·3 |
| Lip | 5 | 4·3 |
| Penis | 4 | 3·4 |
| Pancreas | 4 | 3·4 |
| Bones | 4 | 3·4 |
| Prostate | 3 | 2·6 |
| Rectum | 3 | 2·6 |
| Tongue | 3 | 2·6 |
| Mouth | 2 | 1·7 |
| Nasal sinuses | 2 | 1·7 |
| Lung and bronchus | 2 | 1·7 |
| Colon | 2 | 1·7 |
| Lymph nodes | 2 | 1·7 |

**Johannesburg 1953–55 (Higginson and Oettlé) — Rural, 1953–55**

| Site | Total | % |
|---|---|---|
| Liver | 36 | 20·5 |
| Skin | 25 | 14·2 |
| Rectum | 14 | 8·0 |
| Lung and bronchus | 13 | 7·4 |
| Bones | 11 | 6·3 |
| Prostate | 8 | 4·5 |
| Bladder | 8 | 4·5 |
| Sinuses | 6 | 3·4 |
| Leukaemia | 6 | 3·4 |
| Lymphomata | 6 | 3·4 |
| Kaposi's tumour | 5 | 2·8 |
| Salivary gland | 5 | 2·8 |
| Oesophagus | 4 | 2·3 |
| Stomach | 4 | 2·3 |
| Kidney | 4 | 2·3 |
| Mouth | 3 | 1·7 |
| Testis | 3 | 1·7 |
| Pancreas | 2 | 1·1 |
| Larynx | 2 | 1·1 |
| Breast | 2 | 1·1 |
| Eye | 2 | 1·1 |
| Penis and scrotum | 2 | 1·1 |

**Johannesburg 1953–55 (Higginson and Oettlé) — Urban, 1953–55**

| Site | Total | % |
|---|---|---|
| Liver | 114 | 22·0 |
| Oesophagus | 53 | 10·2 |
| Stomach | 41 | 7·9 |
| Lung and bronchus | 40 | 7·7 |
| Hodgkin's disease | 27 | 5·4 |
| Prostate | 21 | 4·0 |
| Lymph nodes: (secondary) | 20 | 3·8 |
| Leukaemia | 18 | 3·4 |
| Mouth | 18 | 3·4 |
| Bladder | 17 | 3·2 |
| Salivary gland | 15 | 2·8 |
| Brain | 14 | 2·7 |
| Skin | 14 | 2·7 |
| Kaposi's tumour | 11 | 2·1 |
| Pancreas | 10 | 1·9 |
| Penis and scrotum | 9 | 1·7 |
| Sinuses | 9 | 1·7 |
| Larynx | 8 | 1·5 |
| Colon | 7 | 1·3 |
| Bones | 6 | 1·1 |

**Durban 1950–56 (Wainwright)**

| Site | Total | % |
|---|---|---|
| Skin* | 271 | 14·1 |
| Liver* | 128 | 7·2 |
| Oesophagus* | 119 | 6·1 |
| Lung and bronchus* | 89 | 4·5 |
| Prostate | 63 | 3·2 |
| Bladder* | 54 | 2·8 |
| Penis | 54 | 2·8 |
| Mouth, tongue* | 50 | 2·5 |
| Stomach* | 44 | 2·2 |
| Naso-pharynx* | 44 | 2·2 |
| Intestine, colon* | 38 | 1·9 |
| Kaposi's tumour* | 25 | 1·3 |

**Stanleyville 1939–55 (Thijs)**

| Site | Total | % |
|---|---|---|
| Reticulo-endothelial tumours | 190 | 16·3 |
| Liver | 185 | 16·0 |
| Skin | 179 | 15·5 |
| Kaposi's tumour | 155 | 13·3 |
| Sarcoma | 68 | 5·9 |
| Penis | 45 | 3·8 |
| Salivary gland | 33 | 2·9 |
| Stomach | 19 | 1·6 |
| Eye | 19 | 1·6 |
| Breast | 18 | 1·5 |
| Testis | 12 | 1·0 |
| Scrotum | 12 | 1·0 |

**Kampala 1952–53 (Davies)**

| Site | Total | % |
|---|---|---|
| Skin | 69 | 16·3 |
| Liver | 43 | 10·1 |
| Penis | 42 | 10·0 |
| Bladder | 20 | 4·7 |
| Prostate | 19 | 4·5 |
| Stomach | 19 | 4·5 |
| Colon | 15 | 3·5 |
| Eye | 13 | 3·0 |
| Oesophagus | 11 | 2·6 |
| Rectum | 10 | 2·3 |
| Lung and bronchus | 8 | 1·9 |
| Pancreas | 5 | 1·1 |

**Nigeria 1935–44 (Elmes and Baldwin)**

| Site | Total | % |
|---|---|---|
| Liver | 57 | 11·9 |
| Kaposi's tumour | 22 | 4·6 |
| Stomach | 13 | 2·7 |
| Penis | 10 | 2·1 |
| Prostate | 8 | 1·7 |
| Breast | 7 | 1·5 |
| Testis | 7 | 1·5 |
| Scrotum | 5 | 1·0 |

‡ Tumours less than 1·0 per cent are not included in this table.
† Europeans from Lourenço Marques included for comparison.
* Denotes both sexes.

TABLE VIII.—*Order of Frequency of Malignant Neoplasms in African Females in Different Parts of the Continent of Africa* ‡

Lourenço Marques — European†

| Site | Total | % |
|---|---|---|
| Breast | 66 | 23·8 |
| Cervix uteri | 55 | 19·8 |
| Skin | 42 | 15·1 |
| Corpus uteri | 14 | 5·0 |
| Stomach | 13 | 4·7 |
| Sarcoma | 13 | 4·7 |
| Chorion-epithelioma | 10 | 3·6 |
| Ovary | 9 | 3·2 |
| Gall bladder | 7 | 2·5 |
| Vagina | 6 | 2·1 |
| Colon | 6 | 2·1 |
| Hodgkin's disease | 6 | 2·1 |
| Small intestine | 3 | 1·0 |
| Liver | 3 | 1·0 |
| Lymph nodes: (secondary) | 3 | 1·0 |

Lourenço Marques — African

| Site | Total | % |
|---|---|---|
| Cervix uteri | 89 | 20·6 |
| Liver | 63 | 14·6 |
| Skin | 60 | 13·9 |
| Breast | 32 | 7·4 |
| Bladder | 31 | 7·2 |
| Lymphomata | 25 | 5·8 |
| Vagina | 24 | 5·5 |
| Eye | 19 | 4·4 |
| Sarcoma | 15 | 3·4 |
| Bones | 11 | 2·5 |
| Mouth | 9 | 2·1 |
| Lip | 7 | 1·6 |
| Thyroid | 6 | 1·4 |
| Ovary | 5 | 1·1 |
| Salivary gland | 5 | 1·1 |

Johannesburg 1926–33 (Berman)

| Site | Total | % |
|---|---|---|
| Cervix uteri | 27 | 27·3 |
| Breast | 25 | 25·3 |
| Corpus uteri | 15 | 15·2 |
| Ovary | 6 | 6·1 |
| Stomach | 6 | 6·1 |
| Liver | 5 | 5·0 |
| Skin | 3 | 3·0 |
| Thyroid | 3 | 3·0 |
| Lip | 1 | 1·0 |
| Salivary gland | 1 | 1·0 |
| Colon | 1 | 1·0 |
| Rectum | 1 | 1·0 |
| Pancreas | 1 | 1·0 |
| Nose | 1 | 1·0 |
| Vulva | 1 | 1·0 |
| Kidney | 1 | 1·0 |
| Lymph nodes | 1 | 1·0 |

Johannesburg 1953–55 (Higginson and Oettlé)

Rural, 1953–55

| Site | Total | % |
|---|---|---|
| Cervix uteri | 68 | 27·4 |
| Skin | 30 | 12·1 |
| Breast | 28 | 11·3 |
| Liver | 15 | 6·0 |
| Bones | 14 | 5·6 |
| Salivary gland | 12 | 4·8 |
| Corpus uteri | 9 | 3·6 |
| Lymphomata | 9 | 3·6 |
| Stomach | 8 | 3·2 |
| Sinuses | 7 | 2·8 |
| Ovary | 5 | 2·0 |
| Bladder | 5 | 2·0 |
| Adamantinoma | 4 | 1·6 |
| Kidney | 4 | 1·6 |
| Eye | 4 | 1·6 |
| Pharynx | 3 | 1·2 |
| Vulva | 3 | 1·2 |
| Leukaemia | 3 | 1·2 |

Urban, 1953–55

| Site | Total | % |
|---|---|---|
| Cervix uteri | 198 | 41·6 |
| Breast | 50 | 10·5 |
| Liver | 25 | 5·2 |
| Ovary | 19 | 4·0 |
| Stomach | 17 | 3·5 |
| Leukaemia | 16 | 3·3 |
| Lymphomata | 16 | 3·3 |
| Lymph nodes: (secondary) | 13 | 2·7 |
| Brain | 12 | 2·5 |
| Skin | 12 | 2·5 |
| Salivary gland | 12 | 2·5 |
| Large intestine | 9 | 1·8 |
| Thyroid | 7 | 1·4 |
| Lung and bronchus | 7 | 1·4 |
| Rectum | 6 | 1·2 |
| Chorion-epithelioma | 5 | 1·0 |

Durban 1950–56 (Wainwright)

| Site | Total | % |
|---|---|---|
| Cervix uteri | 374 | 19·2 |
| Skin* | 271 | 14·1 |
| Liver* | 128 | 7·2 |
| Oesophagus* | 119 | 6·1 |
| Breast | 113 | 5·8 |
| Lung and bronchus* | 89 | 4·5 |
| Bladder* | 54 | 2·8 |
| Mouth, tongue* | 50 | 2·5 |
| Stomach* | 44 | 2·2 |
| Nasopharynx* | 44 | 2·2 |
| Intestine, colon* | 38 | 1·9 |
| Ovary | 30 | 1·5 |
| Vulva | 25 | 1·3 |
| Kaposi's tumour * | 25 | 1·3 |

Stanleyville 1939–55 (Thijs)

| Site | Total | % |
|---|---|---|
| Cervix uteri | 162 | 17·9 |
| Skin | 120 | 13·2 |
| Breast | 109 | 12·0 |
| Ovary | 77 | 8·8 |
| Reticulo-endothelial tumours | 67 | 7·4 |
| Sarcoma | 44 | 4·9 |
| Salivary gland | 41 | 4·5 |
| Liver | 41 | 4·5 |
| Vulva | 27 | 2·9 |
| Kaposi's tumour | 18 | 1·9 |
| Stomach | 14 | 1·5 |
| Eye | 13 | 1·4 |
| Corpus uteri | 11 | 1·2 |
| Chorion-epithelioma | 10 | 1·1 |
| Mouth | 9 | 1·0 |

Kampala 1952–53 (Davies)

| Site | Total | % |
|---|---|---|
| Cervix uteri | 58 | 18·5 |
| Ovary | 36 | 11·5 |
| Breast | 28 | 9·0 |
| Skin | 25 | 8·0 |
| Corpus uteri | 19 | 6·0 |
| Liver | 13 | 4·1 |
| Colon | 11 | 3·5 |
| Oesophagus | 6 | 2·0 |
| Rectum | 6 | 2·0 |
| Eye | 6 | 2·0 |
| Stomach | 5 | 1·6 |
| Vagina | 4 | 1·2 |
| Vulva | 4 | 1·2 |
| Thyroid | 4 | 1·2 |
| Salivary gland | 1 | 1·2 |

Nigeria 1935–44 (Elmes and Baldwin)

| Site | Total | % |
|---|---|---|
| Breast | 77 | 21·4 |
| Uterus | 68 | 16·4 |
| Ovary | 20 | 5·5 |
| Chorion-epithelioma | 9 | 2·5 |
| Vulva | 7 | 1·9 |
| Vagina | 5 | 1·4 |
| Stomach | 5 | 1·4 |

‡ Tumours less than 1·0 per cent are not included in this table.
† Europeans from Lourenço Marques included for comparison.
* Denotes both sexes.

are more frequent than in any other part of Africa from which information is available. Furthermore, having regard to the imperfections of the statistical information, there is more than suggestive evidence that liver cancer in males and primary carcinoma of the cervix in females still constitute a pan-African problem in such remotely separated regions as Kampala and Johannesburg. The surprisingly high frequency ratio of cancer of the stomach in African males in Johannesburg and of carcinoma of the oesophagus, bronchus and lungs in males in Johannesburg and Durban suggest that an environmental rather than a genetic factor is at play in the aetiology of these cancers.

Although there are many features in common in the frequency ratio of cancer in various parts of Africa, there is also sufficient diversity to suggest that selection of specific areas for more intensive study might provide much valuable information about the possible aetiological factors responsible for the difference in the susceptibility to cancer of the various organs of the body. In view of the rapidly changing socio-economic conditions in Africa, it is urgent that aetiological studies be initiated without delay lest the opportunity be lost of probing the aetiology of those types of tumours which are afflicting populations not only in the western world but also in Africa and in Asia.

### SUMMARY

An analysis has been presented of 1977 malignant neoplasms diagnosed at autopsy and by histological examination and accumulated over a period of 13 years in the Province of Mozambique, Portuguese East Africa. Of these tumours, 1273 were derived from Africans, 580 from Europeans and 124 from other ethnic groups.

Attention was drawn to the high frequency ratio of carcinoma of the liver in African males and to the fact that 73·5 per cent of all malignant neoplasms occurred in four sites, namely, the liver (43·1 per cent), the skin (13·9 per cent), lymphoid tissues (8·6 per cent) and urinary bladder (7·9 per cent). Carcinoma of the oesophagus, stomach, colon and rectum, lung and bronchus were remarkably low in the African as compared with European males living in Mozambique. Carcinoma of the cervix uteri was the commonest tumour (20·6 per cent) in African females and together with carcinoma of the liver (14·6 per cent), skin (13·9 per cent), and bladder (7·2 per cent) was responsible for 56·3 per cent of all malignancies as compared with 36·6 per cent of tumours in comparable sites in European females. Noteworthy was the high frequency of cervical carcinoma as compared with carcinoma of the body of the uterus and the relatively low frequency of breast cancer (7·4 per cent) in African females.

This analysis disclosed that primary carcinoma of the liver was occurring with unusual frequency in young people although the age of maximum incidence could not be established before completion of the rates survey now in progress.

It became evident that in the absence of well established cancer registries, data accumulated from the hospital and routine pathology laboratory could provide a useful guide to the frequency ratio of various cancers in Africa. From a comparison of the data compiled in Lourenço Marques with that derived from other African territories, it was apparent (1) that amongst African males, primary liver cancer and in females, carcinoma of the cervix uteri, with two exceptions were the most frequently occurring malignant neoplasms and (2) that there were notable differences in the frequency ratios of malignant neoplasms in different regions in Africa. The urgency for initiating aetiological studies to explore the basis for these differences was emphasized.

I wish to acknowledge my indebtedness to Professor Joseph Gillman for his stimulating encouragement and helpful advice and criticism during the progress of this work, and to Dr. Christine Gilbert for her invaluable assistance in the preparation of the manuscript.

I wish to thank the National Cancer Association of South Africa for a generous grant which is facilitating the accomplishment of the present study.

### REFERENCES

BERMAN, C.—(1935) *S. Afr. J. med. Sci.*, **1**, 12.
CAMAIN, R.—(1954) *Bull. Mem. de l'Ecole Med et Pharm. Dakar*, **2**, 208.
DAVIES, J. N. P.—(1948) *E. Afr. med J.*, **25**, 117.
DES LIGNERIS, M. J. A.—(1936) *S. Afr. med. J.*, **10**, 478.
ELMES, B. G. T. AND BALDWIN, R. E. T.—(1947) *Ann. trop. Med. Parasit.*, **41**, 321.
FINDLAY, G. M.—(1949) *J. R. micr. Soc.*, **69**, 166.
GELFAND, M.—(1948) 'The Sick African'. Capetown (Stewart).
HIGGINSON, J.—(1951) *Cancer*, **4**, 1224.
PIRIE, J. H.—(1921) *Med. J. S. Afr.*, **17**, 87.
PRATES, M.—(1938) 'Actas do 1° Cong. Med. de Lourenço Marques'. Set.
SMITH, E. C. AND ELMES, B. G. T.—(1934) *Ann. trop. Med. Parasit.*, **28**, 461.
STRACHAN, A. C.—(1934) *J. Path. Bact.*, **39**, 209.
THIJS, A.—(1957) *Ann. Soc. belge Med. trop.*, **37**, 483.
VINT, F. W.—(1935) *Lancet*, ii, 628.
WAINWRIGHT, J. AND ROACH, G. G.—(1957) *S. Afr. Cancer Bull.*, **1**, 162.


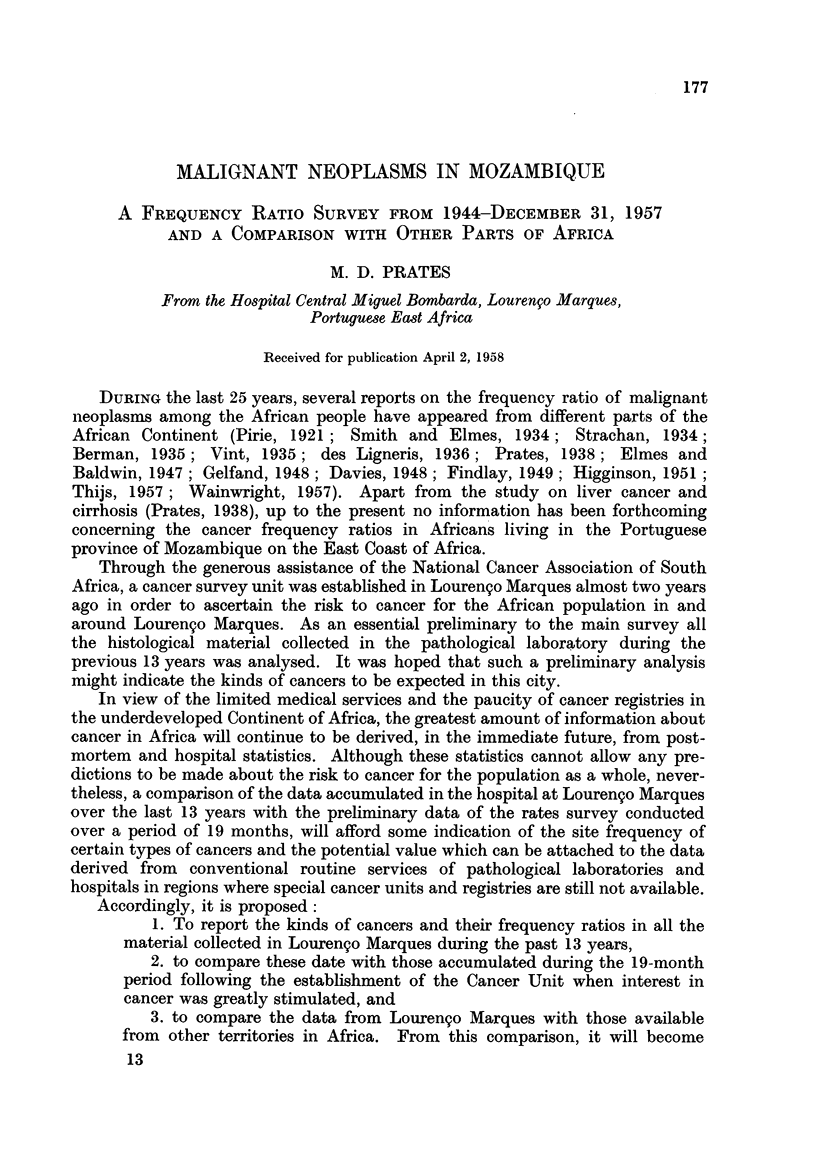

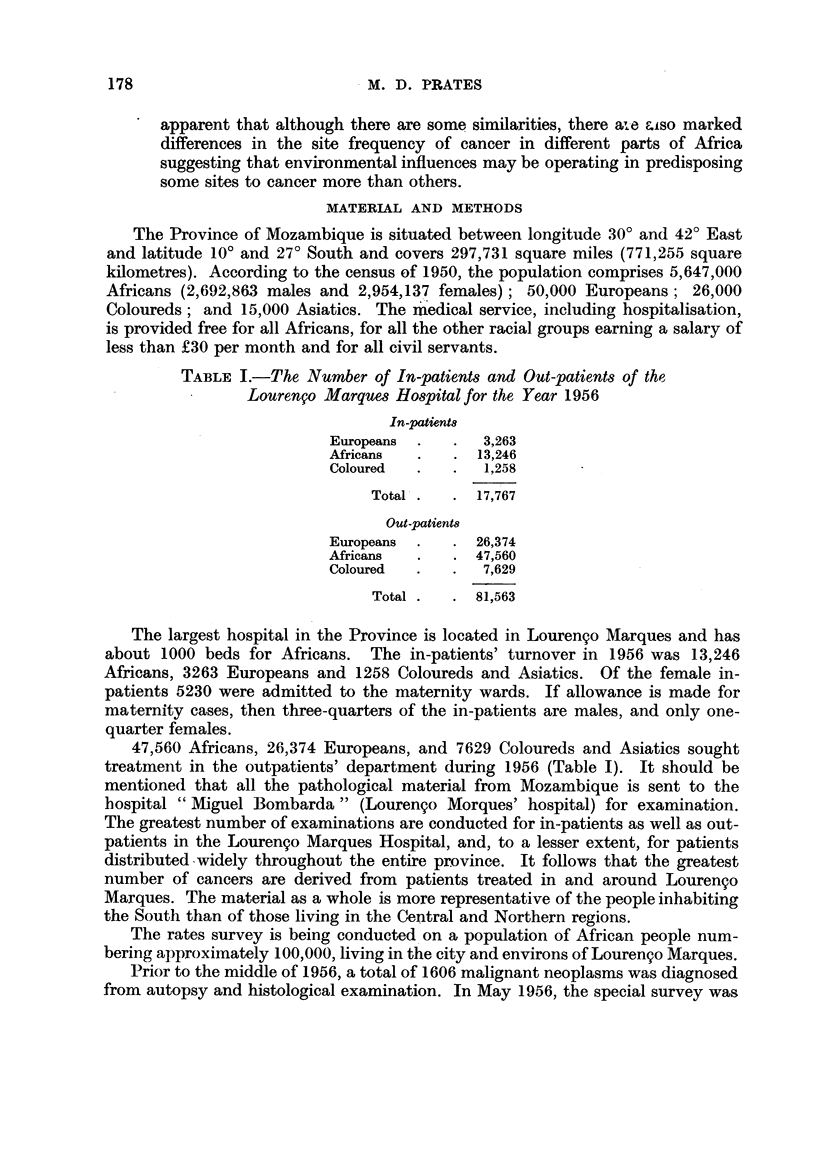

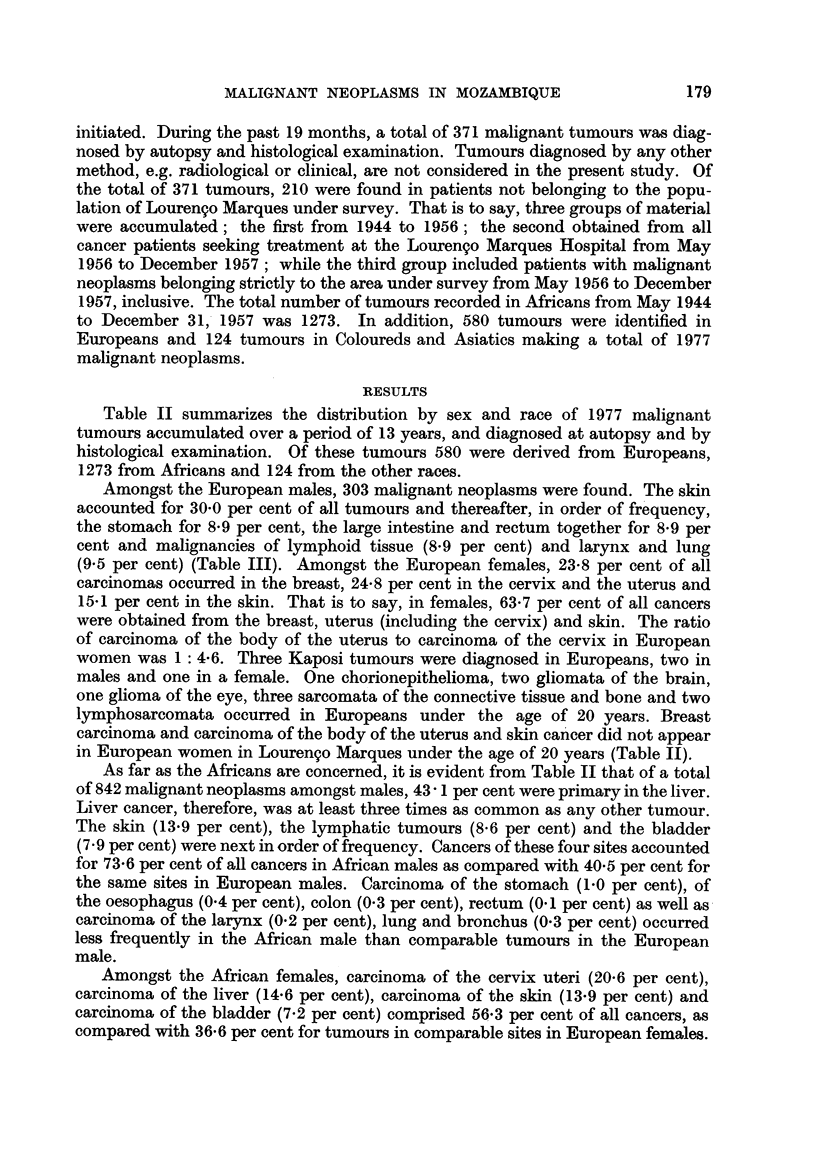

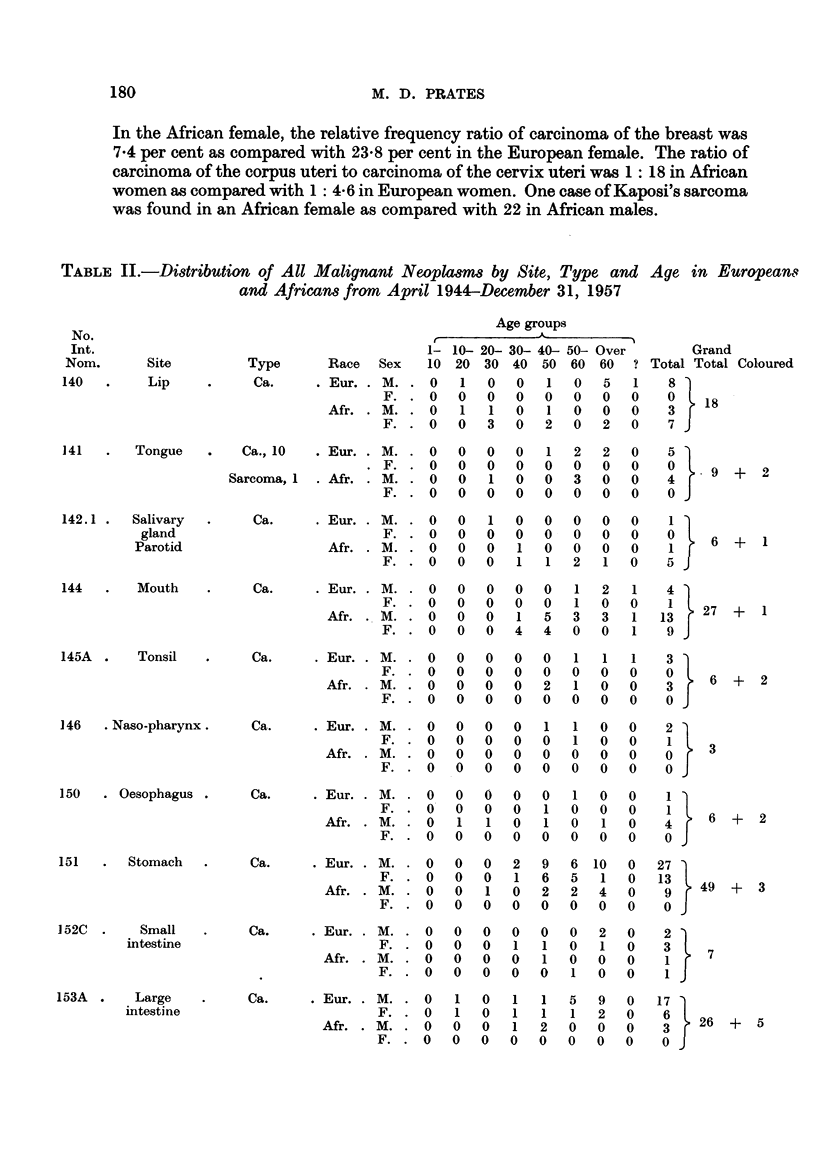

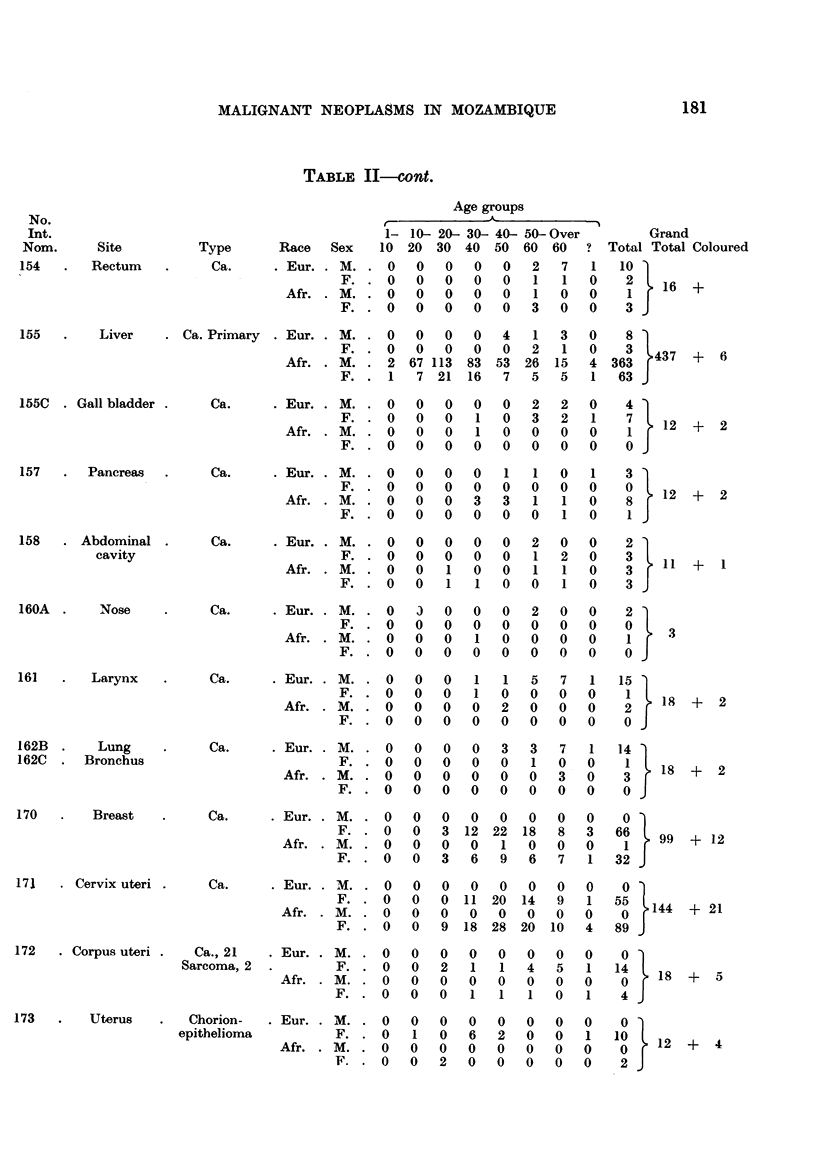

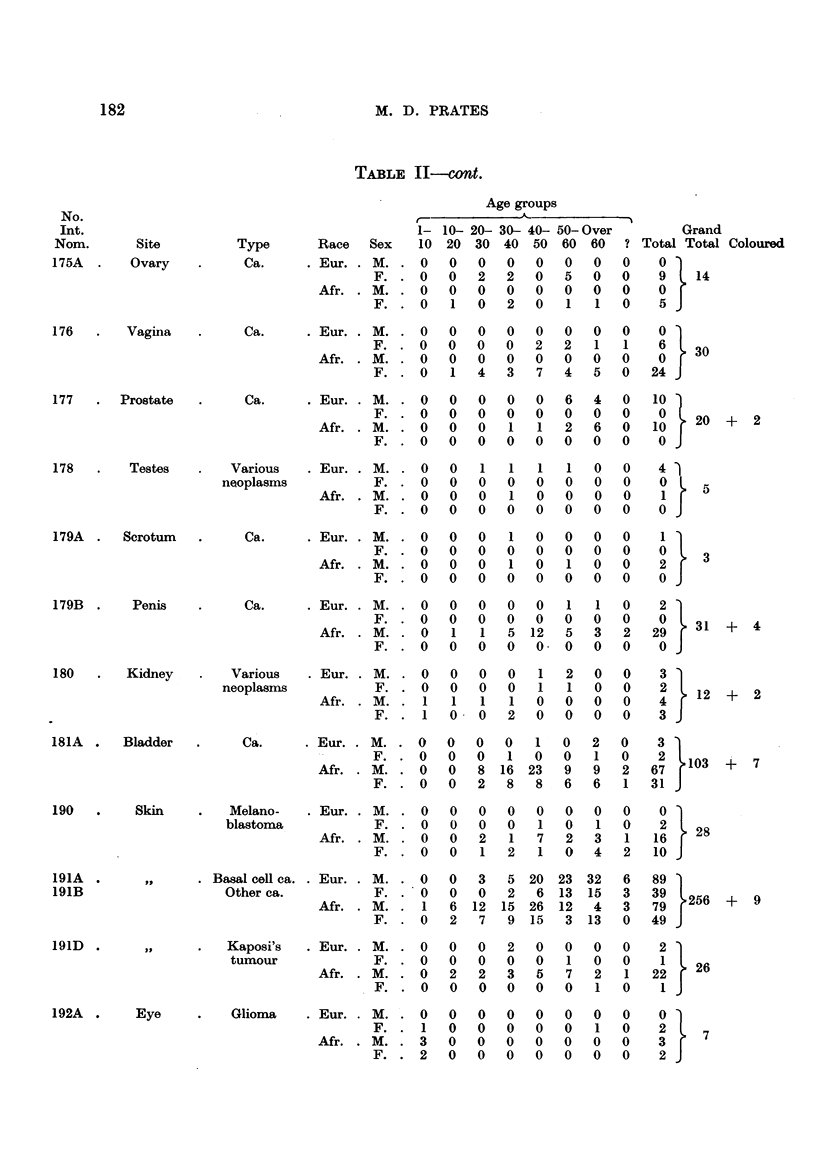

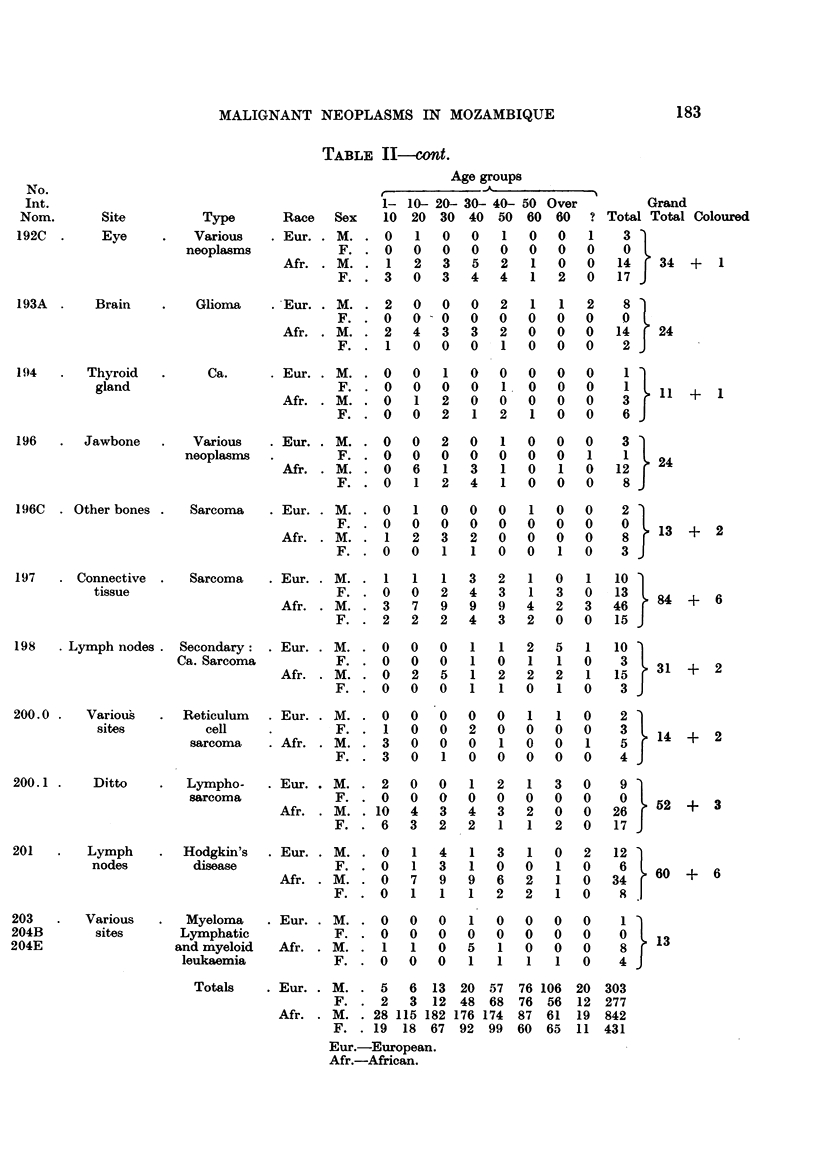

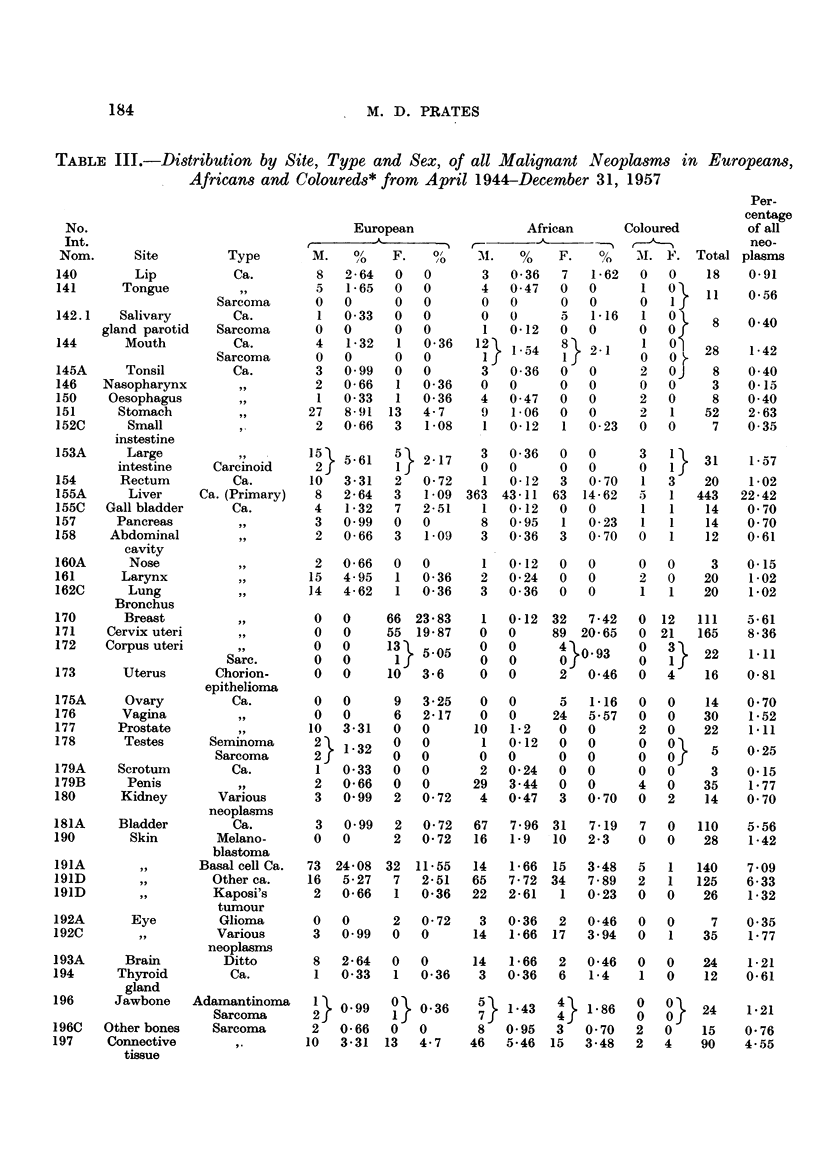

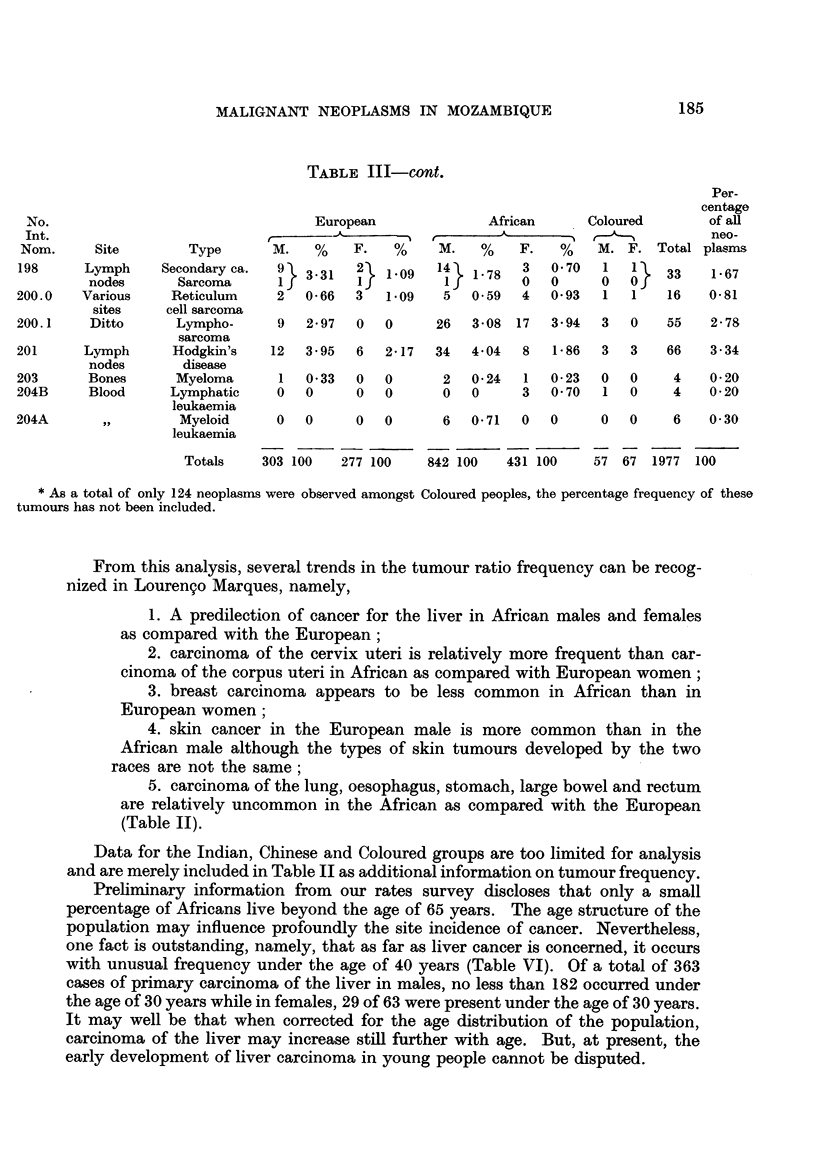

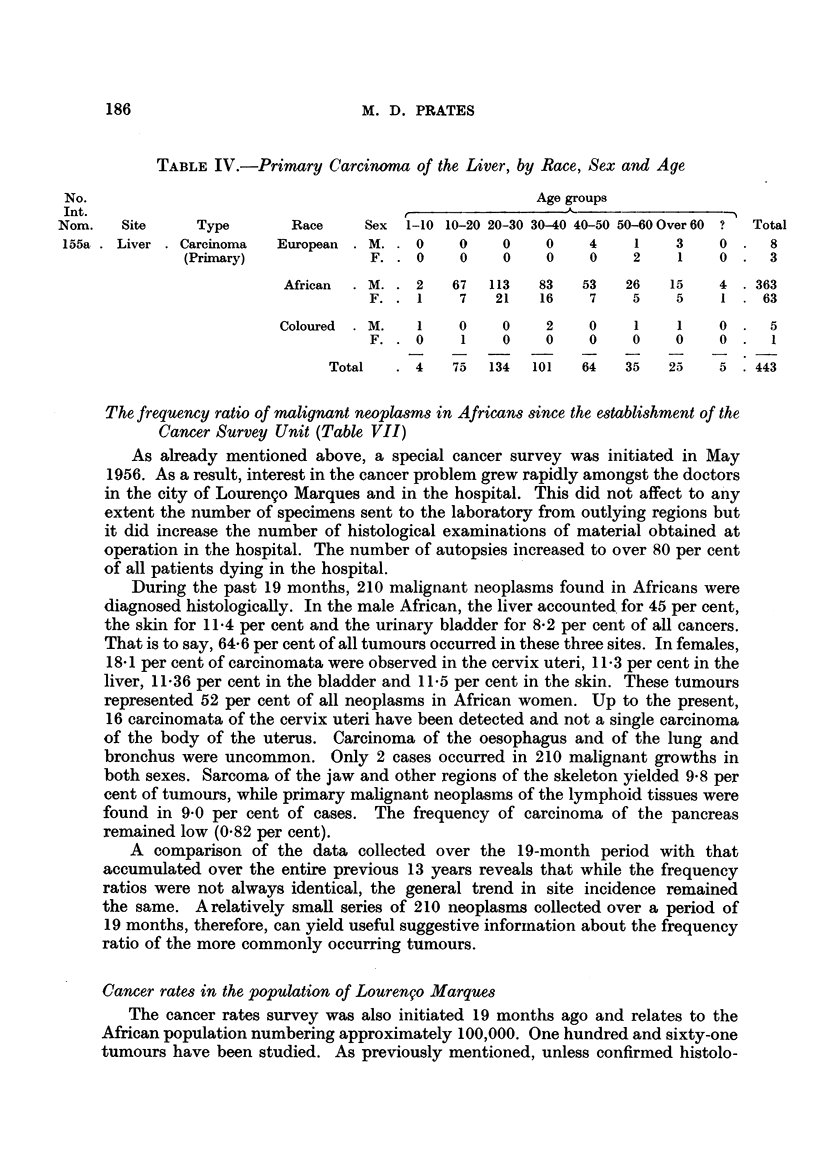

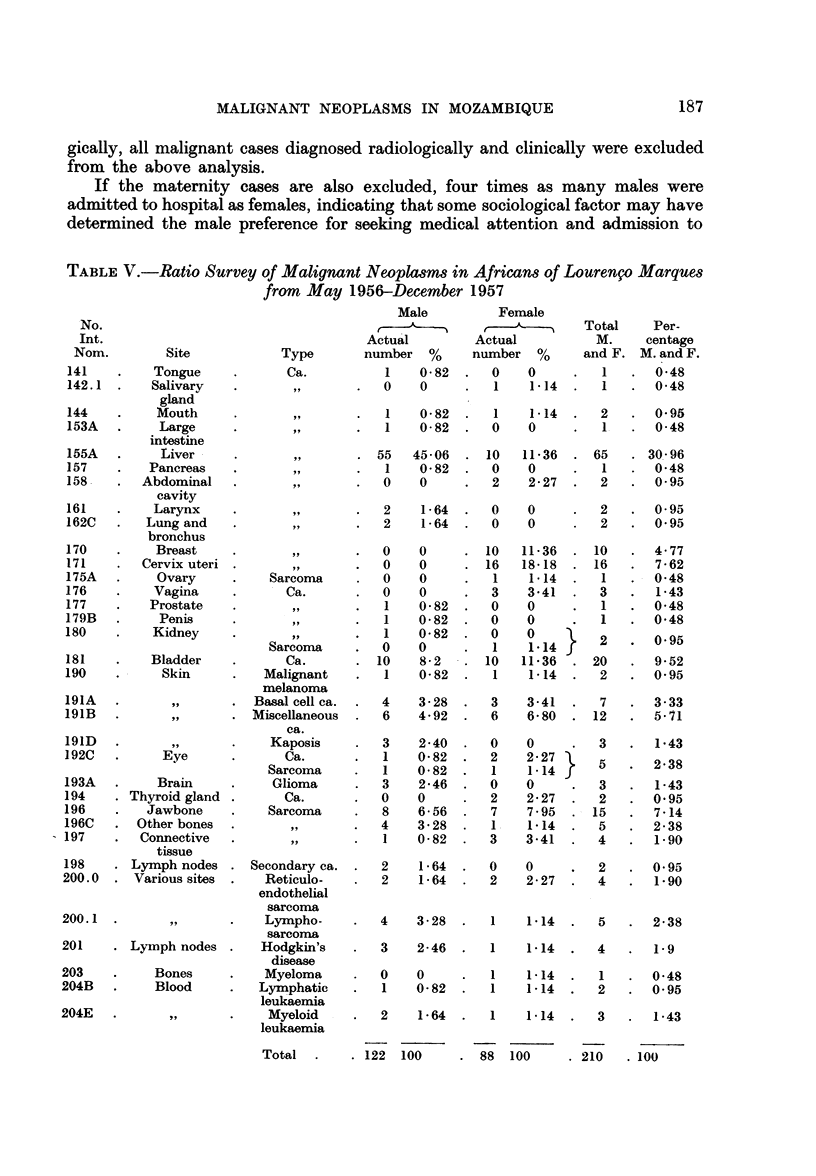

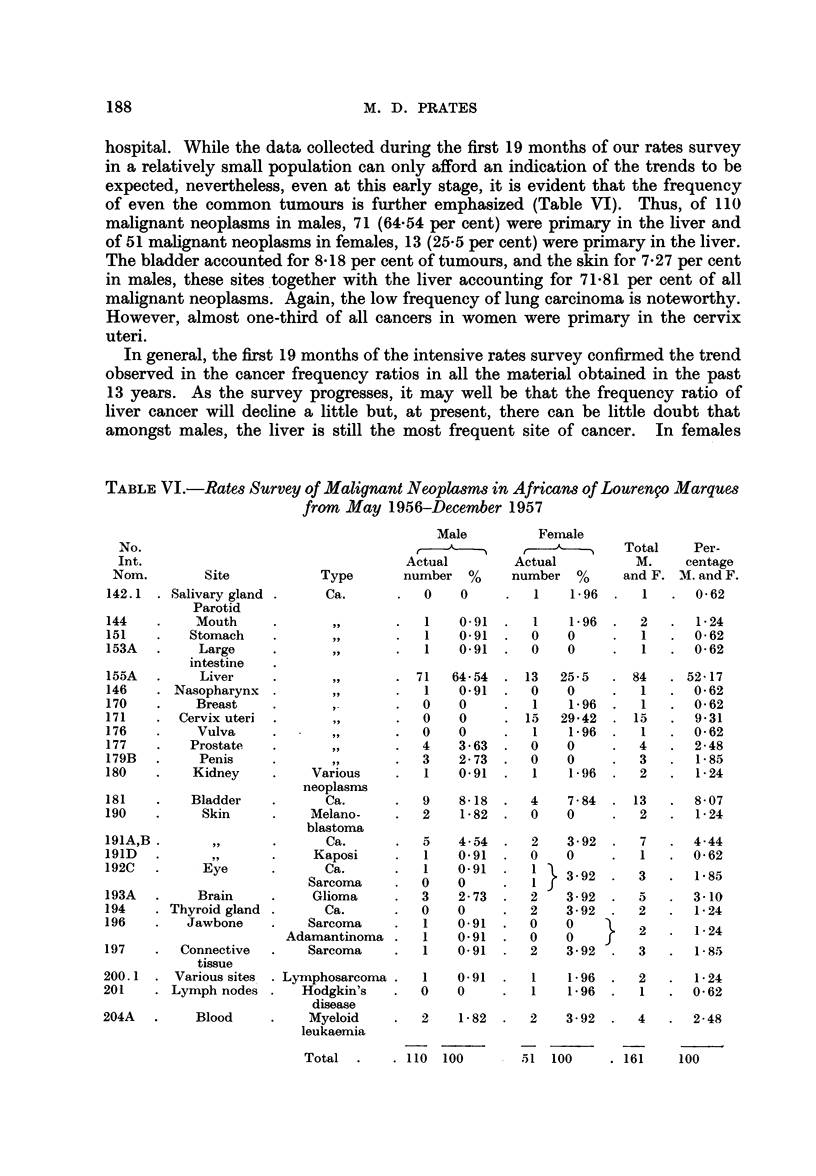

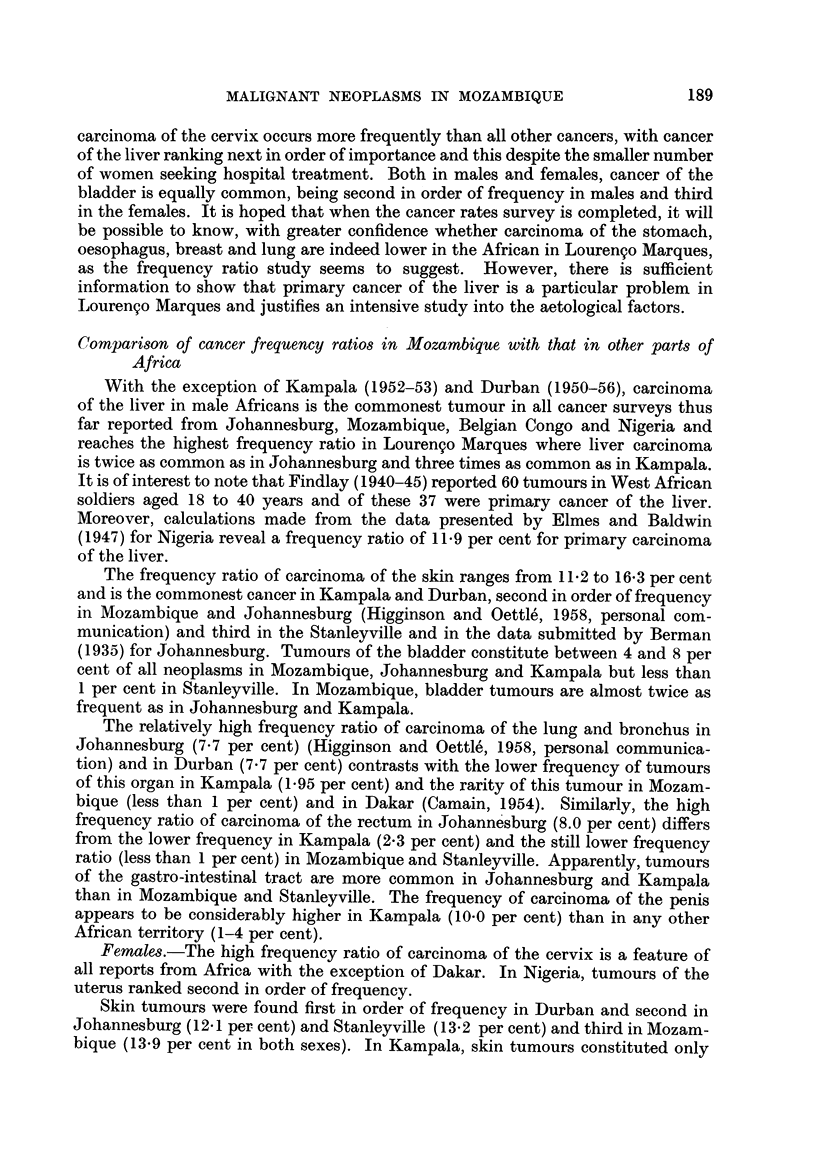

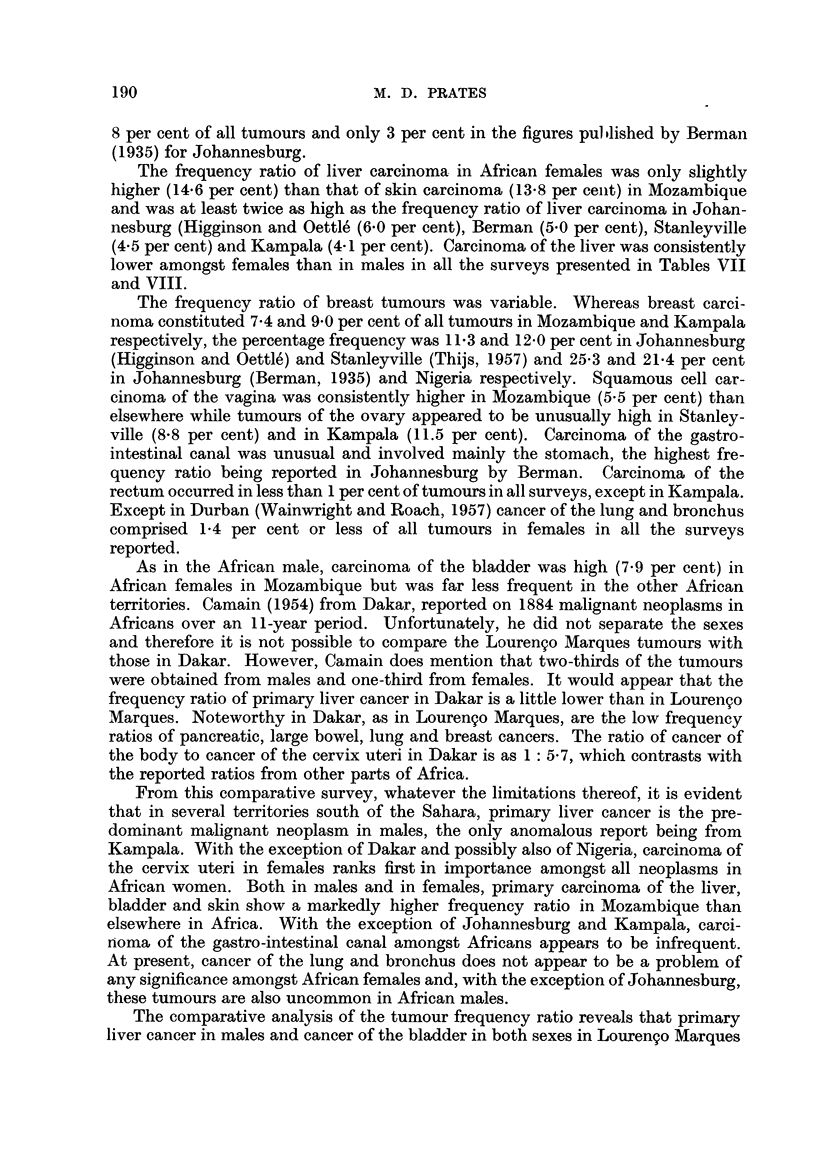

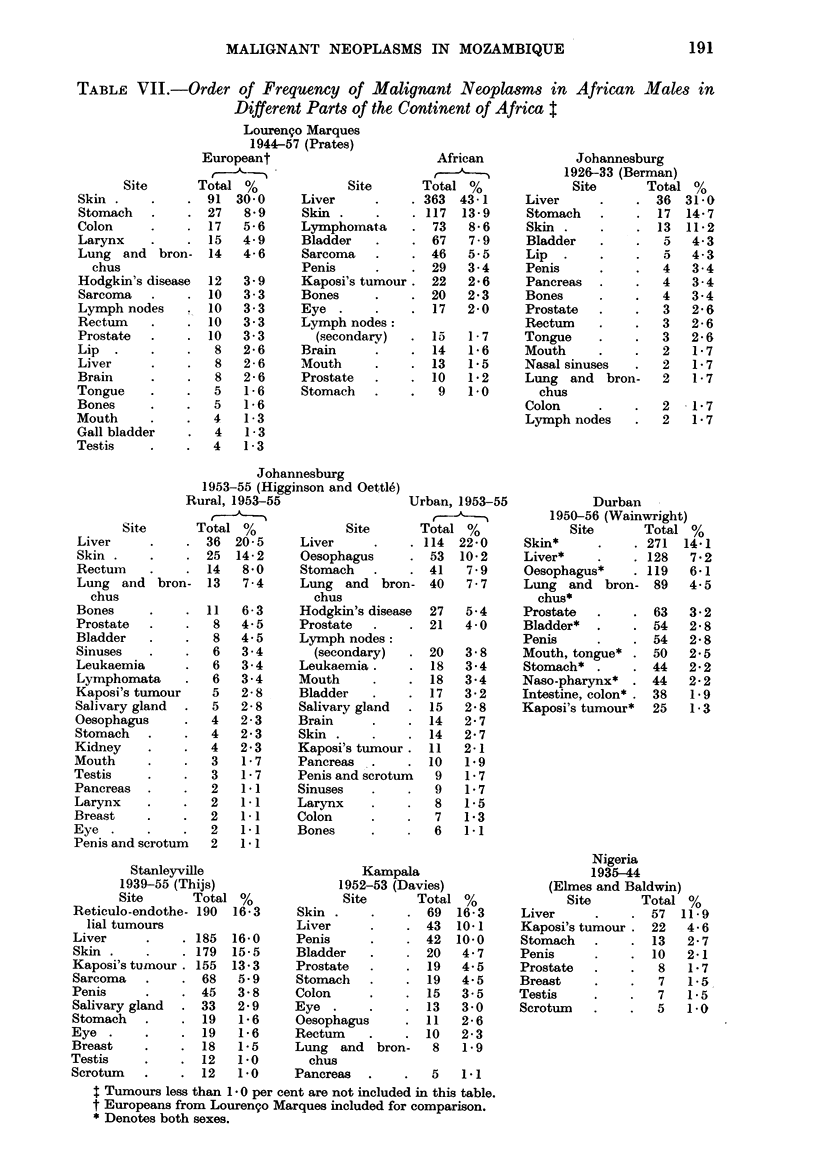

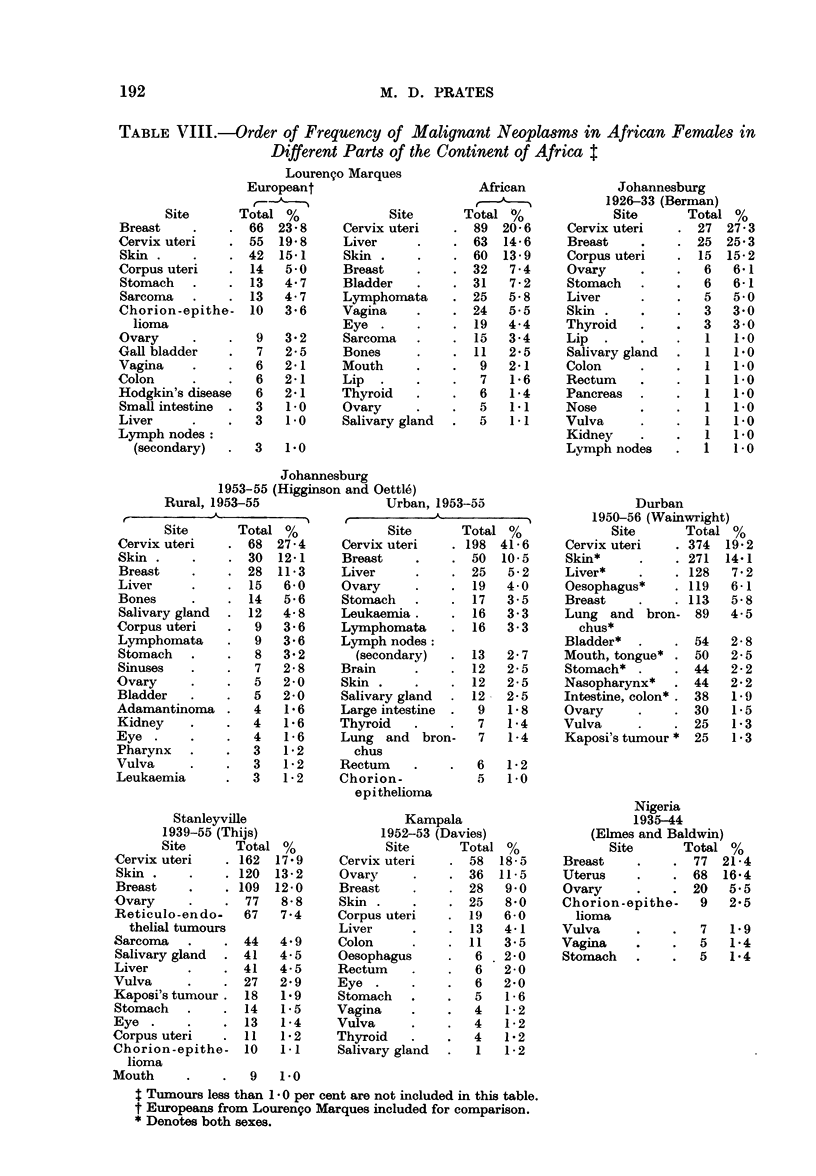

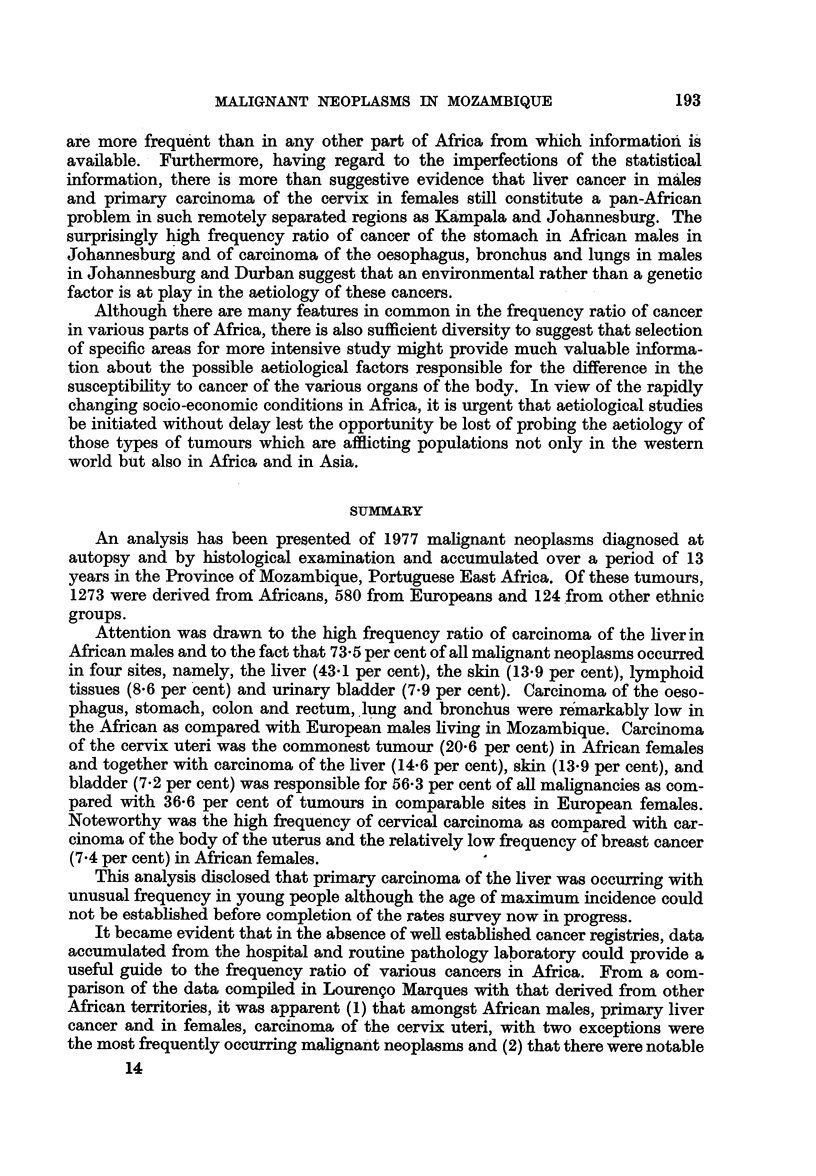

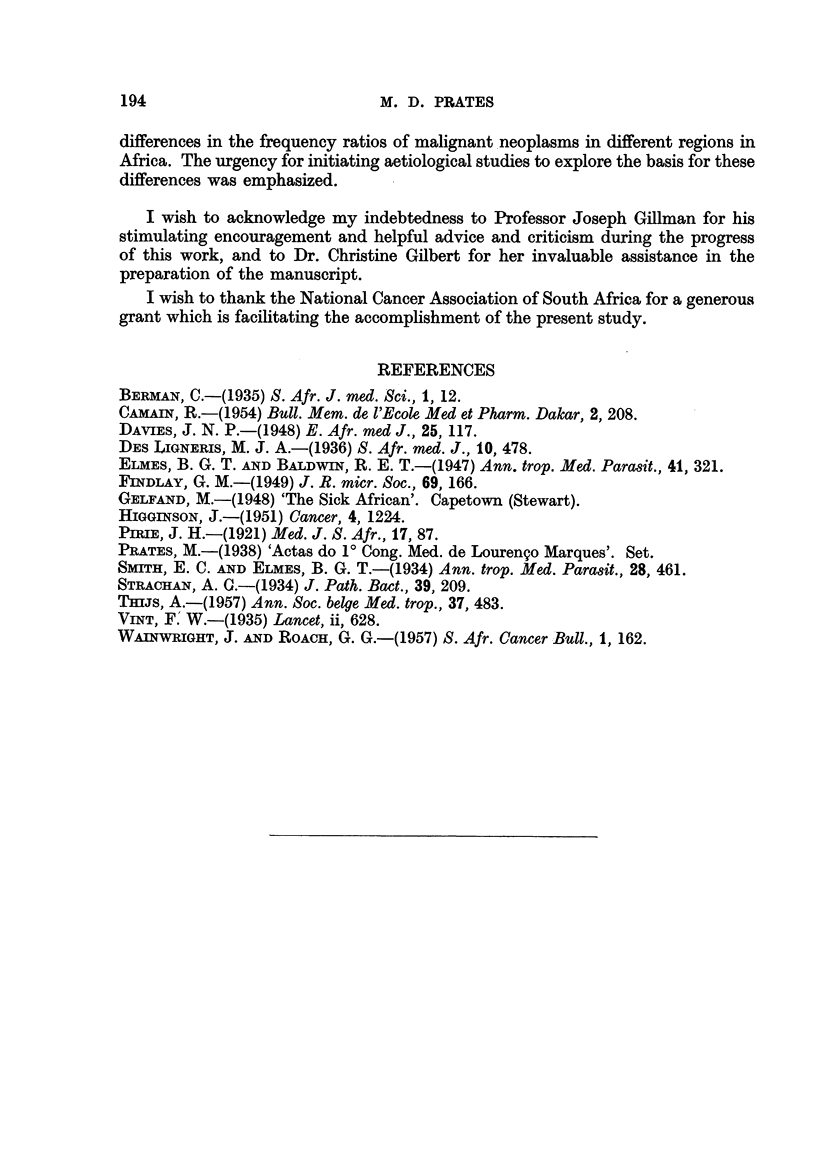


## References

[OCR_03658] HIGGINSON J. (1951). Malignant neoplastic disease in the South African Bantu.. Cancer.

